# Allometric Relationships for Predicting Aboveground Biomass and Sapwood Area of Oneseed Juniper (*Juniperus monosperma*) Trees

**DOI:** 10.3389/fpls.2020.00094

**Published:** 2020-02-26

**Authors:** Andrew M. Cunliffe, Cameron D. McIntire, Fabio Boschetti, Katherine J. Sauer, Marcy Litvak, Karen Anderson, Richard E. Brazier

**Affiliations:** ^1^ Department of Geography, University of Exeter, Exeter, United Kingdom; ^2^ Department of Biology, University of New Mexico, Albuquerque, NM, United States; ^3^ Department of Natural Resource Management, Sul Ross State University, Alpine, TX, United States; ^4^ Environmental Sustainability Institute, University of Exeter, Penryn, United Kingdom

**Keywords:** allometry, sapwood area, carbon stocks, semiarid, remote sensing, woody plant encroachment, bioenergy, brush management

## Abstract

Across the semiarid ecosystems of the southwestern USA, there has been widespread encroachment of woody shrubs and trees including *Juniperus* species into former grasslands. Quantifying vegetation biomass in such ecosystems is important because semiarid ecosystems are thought to play an important role in the global land carbon (C) sink, and changes in plant biomass also have implications for primary consumers and potential bioenergy feedstock. Oneseed juniper (*Juniperus monosperma*) is common in desert grasslands and pinyon–juniper rangelands across the intermountain region of southwestern North America; however, there is limited information about the aboveground biomass (AGB) and sapwood area (SWA) for this species, causing uncertainties in estimates of C stock and transpiration fluxes. In this study, we report on canopy area (CA), stem diameter, maximum height, and biomass measurements from *J. monosperma* trees sampled from central New Mexico. Dry biomass ranged between 0.4 kg and 625 kg, and cross-sectional SWA was measured on n = 200 stems using image analysis. We found a strong linear relationship between CA and AGB (r^2^ = 0.96), with a similar slope to that observed in other juniper species, suggesting that this readily measured attribute is well suited for upscaling studies. There was a 9% bias between different approaches to measuring CA, indicating care should be taken to account for these differences to avoid systematic biases. We found equivalent stem diameter (ESD) was a strong predictor of biomass, but that existing allometric models underpredicted biomass in larger trees. We found SWA could be predicted from individual stem diameter with a power relationship, and that tree-level SWA should be estimated by summing the SWA predictions from individual stems rather than ESD. Our improved allometric models for *J. monosperma* support more accurate and robust measurements of C storage and transpiration fluxes in *Juniperus*-dominated ecosystems.

## Introduction

There is increasing scientific and societal interest in the spatial and temporal dynamics of dryland vegetation. This attention has been motivated by growing recognition of the role semiarid ecosystems may play in controlling interannual variation and longer-term trends in the global land carbon (C) sink (Strand et al., 2008; Poulter et al., 2014; Ahlström et al., 2015; Sleeter et al., 2018). There is also interest in the potential for dryland vegetation to provide bioenergy feedstock (Ansley et al., 2012; Lauer et al., 2015; Chen et al., 2016) and linkages between biomass and landscape fire dynamics (Romme et al., 2009; Archibald et al., 2018) alongside interactions of vegetation cover and land degradation processes (Hochstrasser et al., 2014; Puttock et al., 2014; Cunliffe et al., 2016b). Across the southwestern USA, juniper-dominated and codominant communities are both widespread, and expanding in extent (Manier et al., 2005; Hulet et al., 2014), in part due to human activities (Soulé et al., 2004; Briggs et al., 2007; Van Auken and Smeins, 2008). The native range of *J. monosperma* includes Arizona, New Mexico, Colorado, and the western portions of Texas and Oklahoma (Morin, 1993). Physiologically, *Juniperus monosperma* is among the most drought-tolerant tree species in the southwestern USA, as it has demonstrated the ability to transpire and assimilate C at soil water potential thresholds well below those which are considered typically fatal for other co-occurring species (McDowell et al., 2008a; Gentine et al., 2015; Mackay et al., 2015). The range of this species is therefore expected to expand under a predicted drier climate (Van Auken and Smeins, 2008), although juniper mortality may also increase in the future (Breshears et al., 2005; Gitlin et al., 2006; Kane et al., 2011).

The aboveground biomass (AGB) and associated C storage within juniper-dominated communities is an important fundamental property of these ecosystems. Direct measurement of tree biomass requires destructive harvests, which are both costly and highly disturbing to ecosystems. Consequently, there have been many efforts to develop allometric functions that can be used to estimate AGB nondestructively from dimensional measurements, which have supported significant advances in understanding AGB variability (e.g., Jenkins et al., 2003; Chojnacky et al., 2014; Krofcheck et al., 2016). However, despite this progress, a lack of high-quality observations means that significant uncertainties remain in allometric relationships for many taxonomic groups. The latest review of continental-scale allometric functions in the USA highlighted the relationship between diameter at the root collar (DRC) and AGB for the “woodland Cupressaceae” taxonomic group (consisting of mostly juniper species) as having particularly low confidence due to insufficient observations (Chojnacky et al., 2014). A recent assessment of the C balance of the continental USA also called for more empirical research on the C stocks of non-forest ecosystems (Sleeter et al., 2018). Improving confidence in the allometric relationships underpinning AGB estimates and transpiration fluxes of juniper ecosystems requires new, high-quality observations across the full range of tree sizes.

The stem diameter of *J. monosperma* is notoriously difficult to measure due to its complex branching architecture and dense growth form (Krofcheck et al., 2016). The multi-stem form makes breast-height (1.3 m above ground level) diameter difficult to apply to most *Juniperus* species. Consequently, the diameter at root collar (DRC) is commonly used, with the equivalent stem diameter (ESD) for each tree computed as the sum of all stems measured near ground level (Chojnacky and Rogers, 1999; Chojnacky et al., 2014; Krofcheck et al., 2016). Yet DRC is still time-consuming to measure in the field, thus, there is a need for other forms of allometry that can be applied accurately and preferably inexpensively over larger spatial extents (Strand et al., 2008; Hulet et al., 2014). Canopy area (CA) is a strong predictor of AGB in other species in the *Juniperus* genus (Miller et al., 1981; Tiedemann and Klemmedson, 2000; Sabin, 2008; Ansley et al., 2012) but so far has not been evaluated for *J. monosperma*.

Cross-sectional sapwood area (SWA) is a critical parameter for understanding sapflow through plants (Looker et al., 2016). Sapwood is the hydraulically active portion of the stem responsible for water transport to the living foliage and is a strong predictor of both total leaf area (Vertessy et al., 1995; Köstner et al., 2002; Stancioiu and O'Hara, 2005) and biomass (Morataya et al., 1999; Bond-Lamberty et al., 2002) across many species. SWA is commonly estimated from stem diameter. However, this diameter–SWA relationship is particularly challenging for *Juniperus* species (McDowell et al., 2008b), and there have been only limited investigations for *J. monosperma* using a small number (n ≤ 24) of relatively small stems (≤20 cm diameter) (McDowell et al., 2008b; Pangle et al., 2015). Thus, there is a need to improve knowledge of diameter–SWA relationships that can be used for scaling physiological attributes of this species across the full range of tree sizes that are encountered in mature stands.

This study facilitates measurement of AGB and eco-physiological processes in juniper-dominated rangeland ecosystems. For *J. monosperma*, there has been minimal empirical work describing the relationship between stem diameter and AGB (Grier et al., 1992; Jenkins et al., 2003; Chojnacky et al., 2014), no investigations into the relationship between CA and AGB, and limited information relating stem diameter to SWA (McDowell et al., 2008b; Pangle et al., 2015). We address these knowledge gaps by investigating the relationships between stem diameter, CA, maximum height, SWA, and AGB for *J. monosperma*.

## Methods

### Study Site

We conducted this study in ca. 3 ha area of juniper-savanna, occupied by *J. monosperma* (oneseed juniper) and C4 perennial grasses of which the dominant species is *Bouteloua gracilis* (blue grama). The site is located in the Southwestern Tablelands in the central part of New Mexico, at 34.429°N, 105.861°W, at an elevation of 1,930 m a.s.l. The site is effectively flat with a gradient of <1%. The mean annual precipitation is 364 mm year^-1^, and mean annual temperature is 11.5°C (1987–2017; climate data from PRISM Climate Group, Oregon State University, http://www.prism.oregonstate.edu/). Soils are classed as a well-drained Tapia–Dean loam, derived from alluvium parent material (NRCS, 2018). The site is on private ranchland and has been intermittently grazed by cattle since 1910 (Humphries, 2010). Our harvest site was situated ca. 400 m northeast of the US–WJSAmeriFlux eddy covariance tower (Litvak, 2007), downwind of the tower relative to the prevailing wind direction to avoid disturbance within the footprint. Observations from the flux tower between 2008 and 2017 suggest that this ecosystem is a net sink of C, with net ecosystem exchange net ecosystem exchange of -124.9 ± 6.1 g C m^2^ a^-1^ (where ± is standard error). Biomass sampling occurred in mid-October 2018, concurrent with the seasonal peak mass, with additional stem harvesting for SWA investigation in May 2019. For further discussion on the C dynamics of this site, see Anderson-Teixeira et al. (2011) and Biederman et al. (2017).

### Tree Selection

We selected 20 *J. monosperma* trees for our intensive analysis, encompassing a distribution of sizes that included representatives of the largest individuals observed near the flux tower, smaller saplings, and intermediately sized trees. Our focus was on isolated trees, instead of clusters whose canopy properties might be influenced by adjacent trees, and photographs of these individuals are included in **Table S4**. To supplement our analysis of SWA relationships for larger stem diameters, we also collected an additional 10 large (20–30 cm diameter) stem cross sections.

### Maximum Plant Height

To measure the maximum height (H_Max_) of each individual, we used a GS08Plus Leica Geosystems real-time kinematic global navigation satellite system (RTK-GNSS) instrument to measure the highest point of each plant and the ground surface at four corners around individual harvest plots. This RTK-GNSS has a precision of ca. 15 mm relative to a local reference station, and H_Max_ was calculated as the difference in elevation between the maximum plant height and a terrain surface interpolated between the four corners with inverse distance weighting (using a power of 2). The low-relief, planar topography of the site meant that the interpolated surface provided a good representation of the true terrain height.

### Canopy Area

The selected individuals were surveyed using a lightweight consumer unmanned aerial vehicle (UAV) to acquire aerial images on October 6, 2018. The survey protocol is described in detail in Cunliffe and Anderson (2019). Briefly, surveys were flown using a DJI Phantom 4 Advanced multi-rotor equipped with a built-in CMOS sensor at 9 mm focal length capturing 20M effective pixels. Two sets of survey flights were undertaken, the first obtaining nadir imagery at ca. 21 m above ground level (a.g.l.) and second obtaining oblique (ca. 20° from nadir) images at ca. 25 m a.g.l. Both survey flights obtained 75% forward and side overlap, together capturing at least 34 images for each part of the study area. Camera aperture was f5, sensitivity (ISO) was 200, shutter speed was faster than 1/800 of a second, and images were underexposed by 1/3 of a stop to optimize image quality. UAV operations were undertaken by certified operators using established protocols (Cunliffe et al., 2017) as per Part 107 of the U.S. Federal Aviation Authority regulations. To constrain the photogrammetric reconstruction spatially, we deployed thirteen 20 cm × 20 cm ground control markers across the survey area and geolocated these using the RTK-GNSS.

Aerial images were processed using structure-from-motion photogrammetry using a workflow based on Cunliffe et al. (2016a). Geotagged image data and marker coordinates were imported into Agisoft PhotoScan (v1.4.3) and converted to a common coordinate reference system (EPSG:26913). Image sharpness was assessed using PhotoScan's image quality tool, and all images had a sharpness score of ≥0.84. Photos were matched and cameras aligned using the highest quality setting, key point limit of 40,000, tie point limit of 8,000, generic and reference pair preselection enabled, and adaptive camera model fitting disabled. Reference settings: camera location accuracy = XY ± 20 m, Z ± 50 m; marker location accuracy = XY ± 0.02 m, Z ± 0.05 m; marker projection accuracy was set to two pixels; tie point accuracy was set to one pixel. The sparse cloud was filtered, and points with reprojection error above 0.45 were excluded from further analysis. An operator reviewed the sparse point cloud and estimated camera positions to verify their plausibility. All images were aligned and used for further processing. Geolocated markers were placed by an operator on 10 projected images for each of the 13 ground control points. Three markers used for independent accuracy assessment were deselected at this stage. The bundle adjustment was optimized using the filtered point cloud with the following lens parameters: focal length (f), principal point (cx, cy), radial distortion (k1, k2), tangential distortion (p1, p2), aspect ratio and skew coefficient (b1, b2). Multi-view stereopsis (dense point cloud generation) was undertaken using the ultrahigh-quality setting, mild depth filtering, and calculate point colors enabled. A triangular irregular network (TIN) was built from the dense point cloud *via* Delaunay triangulation using a high face count (⅕ the number of points in the dense cloud) and height field approach with interpolation enabled. An orthomosaic was built at the native spatial resolution, projected onto the TIN surface (“model data”) using the “mosaic” blending mode with “fill holes” enabled. The orthomosaic was exported as uncompressed GeoTIFF at a spatial grain of 0.01 m.

The orthomosaic was loaded into a GIS (ESRI ArcPro v2.1.3), and the edge of each individual's canopy was manually digitized by the same operator at a scale of 1:40. The area (in square meters) of each canopy polygon was extracted (*CA_1_*), and the length (in meters) of the widest (*a*) and perpendicular (*b*) canopy diameters were extracted using the minimum bounding geometry utility in ArcPro. To test the (dis)similarity between CA derived directly from polygons (*CA_1_*) against CA derived from typical field observations (*CA_2_*), CA was also calculated as follows:

(1) $$CA_{2}=\pi \;/\;4\;\left(a\;b\right)$$

### Biomass Measurement

Individual trees were harvested over 2 days (October 8 and 9, 2018). Logistical constraints with harvesting larger individuals meant that only 18 of the 20 individuals selected for intensive study could be harvested. Biomass from each tree was separated into two components, (i) wood tissue (>3 cm stem diameter, including any bole wood) and (ii) leaf and twig tissue (<3 cm diameter, hereafter termed “leaf/twig”; Tiedemann and Klemmedson, 2000; Ansley et al., 2012). Trees were harvested to the ground line, and the wet weights of both components were measured within 2 h of each tree being felled. Subsamples of the wood and leaf/twig components for each component of each tree were collected, weighed to determine wet weight, and oven-dried at 80°C for ≥93 h to a constant weight (defined as <0.1% change in mass in 24 h) to determine their water content. These dried subsamples were up to 18 kg, accounting for at least 1.6% and up to 100% of each tree. Water contents were calculated on a green weight basis for each tree component, i.e. (wet mass - dry mass)/wet mass, and were used to convert wet mass to dry mass. The component proportions were calculated on a dry-weight basis. AGB was calculated as the sum of wood tissue and leaf and twig tissue for each individual.

### Basal and Sapwood Area

The DRC was defined as the lowest primary branching point for a single stem, where a stem either intersected with a main bole or with the ground (Grier et al., 1992; Chojnacky and Rogers, 1999; Krofcheck et al., 2016). DRC_Wet_ was measured in the field for all disks >5 cm using a thin-line diameter tape and for all disks <5 cm using the mean of two measurements about the major and minor axes using a digital caliper. We obtained n = 200 stem cross sections (hereafter “disks”) from a total of n = 20 individual trees at DRC. These disks were air-dried over ≥30 days (to minimize cracking) and were then remeasured to determine the DRC_Dry_. Dried disks were smoothed using a combination of belt and orbital sanders at progressively finer grits to clearly distinguish the sapwood–heartwood interface. Fine-resolution images (45.7 Megapixels) were taken for each disk using a DSLR camera (Nikon D850). The SWA of each disk was measured using the image processing software ImageJ (Schneider et al., 2012), scaled using known length in each image, with visual differentiation of the sapwood–heartwood interface, and reported on a whole tree basis. An inferred DRC measurement was also back-calculated from the imaged basal area (BA) measurements as:

(2) $$DRC=\;\sqrt{\frac{BA}{\pi }}\times 2$$

The ESD for each tree was calculated as the square root of the sum of the squared DRC for all of the stems for that individual, for both wet and dry diameter measurements (Krofcheck et al., 2016). ESD includes bark thickness, so that it could be measured non-destructively. BA was calculated from wet and dry ESD using:

(3) $$BA_{Wet}=\pi \cdot {\left(\frac{ESD_{Wet}}{2}\right)}^{2}\;$$

(4) $$BA_{Dry}=\pi \cdot {\left(\frac{ESD_{Dry}}{2}\right)}^{2}$$

where BA is in square centimeters, and ESD is equivalent DRC in centimeters, thus allowing for comparison of imaged ESD with measurements of wet and dry ESD. We did not include age estimates of sampled stems because they could not be determined reliably for this taxon. Juniper has highly variable growth patterns, making it very difficult to count annual growth rings reliably (**Figure S1**), and juniper is prone to the production of false rings (two or more rings per year) and missing rings (due to particularly poor growing conditions). Together, these issues prevent the validation of dendrochronological estimates by cross-dating observations between different *J. monosperma* stems (Grissino-Mayer, 1993).

### Carbon and Nitrogen Content

We determined total C and total nitrogen contents for the <3 cm and, where present, > 3 cm components of n = 12 individual plants. Representative subsamples were taken from each partition, incorporating minimal bark in the >3 cm partition and including leaf and twig issue in the <3 cm partition. Subsamples of oven-dried biomass were ground to a fine powder using a high-energy ball mill (Retsch Mixer Mill MM400), and 5-mg aliquots analyzed using flash combustion in an elemental analyzer (Flash 2000; Thermo Scientific).

### Statistical Analysis

Statistical analysis was undertaken in R (v3.6.0, R Core Team, 2019). We compared the diameters of wet versus dry disks with a one-way, paired-samples t-test. Nonlinear models were fitted with the nonlinear least-squares function using the Gauss–Newton algorithm. Results were visualized using ggplot2 (Wickham, 2018 v3.1.0). We used power models to describe AGB as a function of ESD and maximum height (Ansley et al., 2012) and SWA as a function of stem diameter (Köstner et al., 1998; Pangle et al., 2015; Aparecido et al., 2019). We believe that power models have a stronger biological basis than the logarithmic models previously used to predict biomass from ESD [sensu (West et al., 1997)]. We used linear models for predicting AGB from CA in order to facilitate integration into upscaling studies using remotely sensed estimates of canopy cover (Sabin, 2008; Starks et al., 2011; Ansley et al., 2012; Mirik et al., 2013; Hulet et al., 2014). We computed the 95% confidence interval of the linear CA–biomass models with the “stat_smooth” function in the “ggplot2” package and the 95% prediction intervals for the canopy height and SWA models using the “predictNLS” function in the “propagate” package (Spiess, 2018; v1.0.6). For comparison with published allometric functions for this taxon, we used field observations of wet ESD (ESD_Wet_) to estimate total biomass. Eq. 5 is from Jenkins et al. (2003) for the “woodland” class using the recommended conversion from DRC to diameter at breast height (DBH) after Chojnacky and Rogers (1999), Eq. 6 is from Chojnacky et al. (2014) for the “woodland Cupressaceae” class, and Eq. 7 is from Grier et al. (1992) for *J. monosperma*.

(5) $$M_{Jenkins}=EXP\left(-0.7152+1.7029\;ln\;DBH\right)$$

(6) $$M_{Chojnacky}=EXP\left(-2.709+2.1942\;ln\;ESD_{Wet}\right)$$

(7) $$M_{Grier}=10^{\left(-1.157+2.086\;\;log\;ESD_{Wet}\right)}$$

Where M is the total aboveground dry biomass in kilograms, DBH is diameter at breast height in centimeters, and ESD_Wet_ is equivalent DRC in centimeters.

For comparison with SWA–stem diameter relationships, we used field observations of wet ESD (ESD_Wet_) to estimate SWA with the following equations: Eq. 8 is from McDowell et al. (2008b) and Eq. 9 is from Pangle et al. (2015), both for *J. monosperma* sampled in New Mexico.

(8) $$SWA_{McDowell}=4.3\;\times \;D_{Wet}-9.8$$

(9) $$SWA_{Pangle}=0.8227\;\times \;D_{\text{wet}}^{1.3903}$$

Where SWA is sapwood area in square centimeters, and D_Wet_ is stem diameter in centimeters.

CA was computed from canopy length and width dimensions with Eq. 1, and linear models were fitted with intercepts constrained through the origin. To relate our findings to the existing literature, we also used ordinary least squares regression to fit a linear model to the *J. osteosperma* dataset published by Miller et al. (1981), whose 33 samples ranged in size from 11.7 to 957.3 kg per tree with CAs between 3.3 and 69.9 m^2^.

## Results

The 18 trees harvested had AGB ranging from 0.36 to 625.55 kg per individual and ESDs (ESD_Wet_) ranging from 5.2 to 48.5 cm. Our measurements are reported in **Table S3**, and summary statistics for measured attributes are presented in **Table 1**.

**Table 1:** Statistical summary of measured attributes from the primary set of observed trees.

Attribute		Unit	Minimum	Maximum	Mean	N
AGB	Total	kg	0.36	625.55	110.62	18
	<3	kg	0.36	257.33	51.86	18
	>3	kg	0.00	368.22	58.76	18
Moisture content	Total^1^	%	31.1	61.8	41.6	18
	<3	%	31.5	61.8	43.5	18
	>3	%	24.8	44.8	35.9	18
Maximum height	(H_Max_)	m	0.62	5.83	2.37	20
CA	CA_1_	m^2^	0.09	56.86	13.94	20
	CA_2_	m^2^	0.10	60.34	14.91	20
ESD	ESD_Wet_	cm	5.2	48.5	21.9	15
	ESD_Dry_	cm	3.3	47.2	20.7	15
	ESD_Image_	cm	2.6	48.6	21.1	15

^1^Total moisture content is the mass-weighted value for the whole tree, and AGB is the dry aboveground biomass of each individual.

AGB, aboveground biomass; CA, canopy area; ESD, equivalent stem diameter

### Predictors of Aboveground Biomass

**Figure 1:**
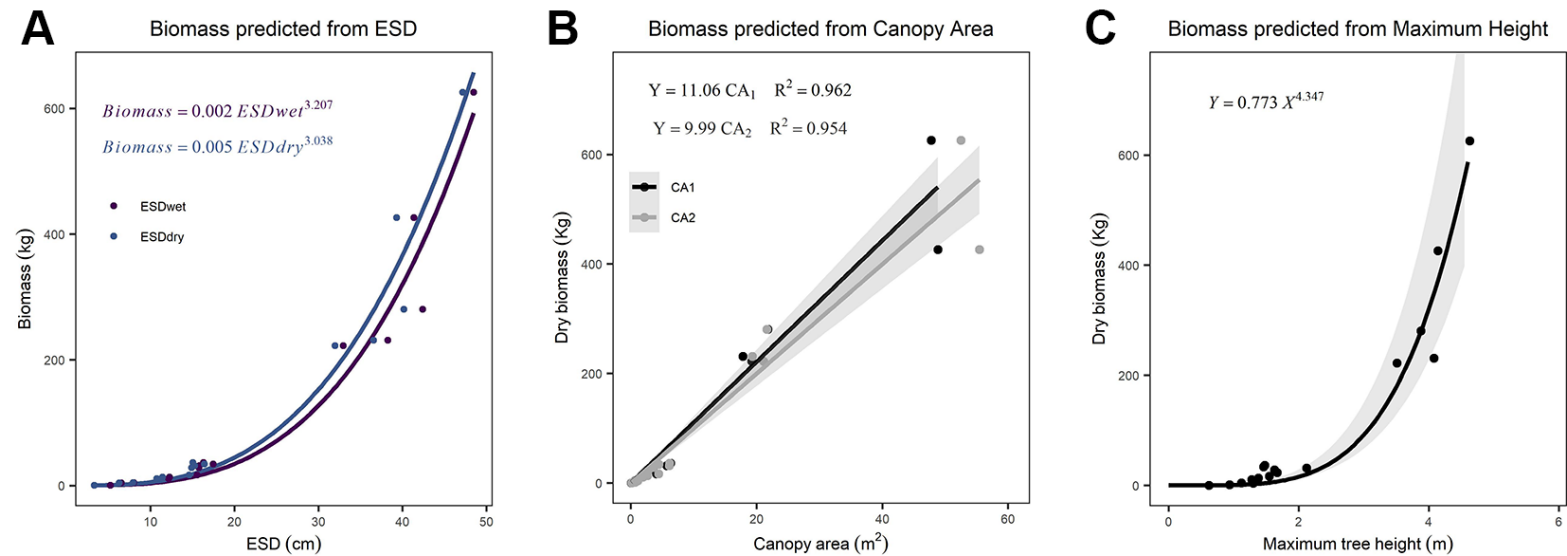
Relationships between explanatory variables and aboveground biomass (AGB) for **(A)** equivalent stem diameter (ESD) using both wet and dry measurements (n = 15), **(B)** canopy area (n = 18), and **(C)** maximum tree height (n = 18). The shaded areas indicate the 95% prediction intervals on panel **(C)** and the 95% confidence interval on **(B)**. Details of all models and parameters are in **Table 2**.

We found ESD, CA, and maximum height were all strong predictors of AGB (**Figure 1**; **Table 2**). The relationship between ESD (both wet and dry measurements) and AGB was described by power models (**Figure 1A**; **Table 2**). The relationship between CA and biomass was linear for both CA_1_ and CA_2_ (**Figure 1B**) and forcing the intercept through the origin made minimal (≤2%) difference to either the model slopes or coefficients of determination (these differ by less than <0.018) (**Table 2**). Power models fitted to the canopy height versus biomass observations had exponents statistically indistinguishable from 1 and also indicate ca. 10–12 kg of biomass per m^2^ of CA (**Table 2**). The more accurate measurements of canopy area (CA_1_) have greater predictive power than CA_2_; however, the difference in coefficient of determination is very small (r^2^ = 0.962 versus 0.954, for CA_1_ and CA_2_, respectively). The relationship between maximum height and AGB was described with a power model (**Figure 1C**).

**Table 2:** Statistical model parameters.

Dependent variable	Predictor variable	Model form	Value of *a* (SE)	Value of *b* (SE)	Overall model significance	R^2^	RMSE	n
Biomass (kg)	ESD_Wet_ (cm)	*Y* = a * X^b^	0.002 (0.004)	3.207 (0.425)	–	–	41.86	15
Biomass (kg)	ESD_Dry_ (cm)	*Y* = a * X^b^	0.005 (0.007)	3.038 (0.352)	–	–	38.55	15
Biomass (kg)	CA_1_ (m^2^)	*y* = a + b * *X*	-8.363 (12.079)	11.329 (0.663)	≤0.001	0.948	41.87	18
Biomass (kg)	CA_1_ (m^2^)	*y* = a + b * *X*	0	11.064 (0.533)	≤0.001	0.962	41.23	18
Biomass (kg)	CA_2_ (m^2^)	*y* = a + b * *X*	-4.438 (13.319)	10.115 (0.663)	≤0.001	0.936	46.59	18
Biomass (kg)	CA_2_ (m^2^)	*y* = a + b * *X*	0	9.989 (0.532)	≤0.001	0.954	45.35	18
Biomass (kg)	CA_1_ (m^2^)	*Y* = a * X^b^	10.972 (4.137)	1.002 (0.102)	–	–	42.49	18
Biomass (kg)	CA_2_ (m^2^)	*Y* = a * X^b^	12.188 (4.777)	0.947 (0.104)	–	–	46.33	18
Biomass (kg)	H_Max_ (m)	*Y* = a * X^b^	0.773 (0.587)	4.347 (0.520)	–	–	37.18	18
SWA (cm^2^)	Stem diameter: all (cm)	*Y* = a * X^b^	1.406 (0.257)	1.407 (0.059)	–	–	15.68	200
SWA (cm^2^)	ESD_Wet_: all (cm)	*Y* = a * X^b^	2.040 (2.517)	1.479 (0.335)	–	–	113.70	15

SE, standard error; n, number of individuals used to fit models; R_2_, coefficient of determination; RMSE, root mean square error; CA_1_, canopy area extracted from polygons; CA_2_, canopy area calculated from two perpendicular measurements of canopy diameter; H_Max_, the maximum canopy height; SWA, sapwood area. Tree-level SWA should preferentially be calculated as the sum of stem-level predictions rather than from ESD; see text for details.

**Figure 2:**
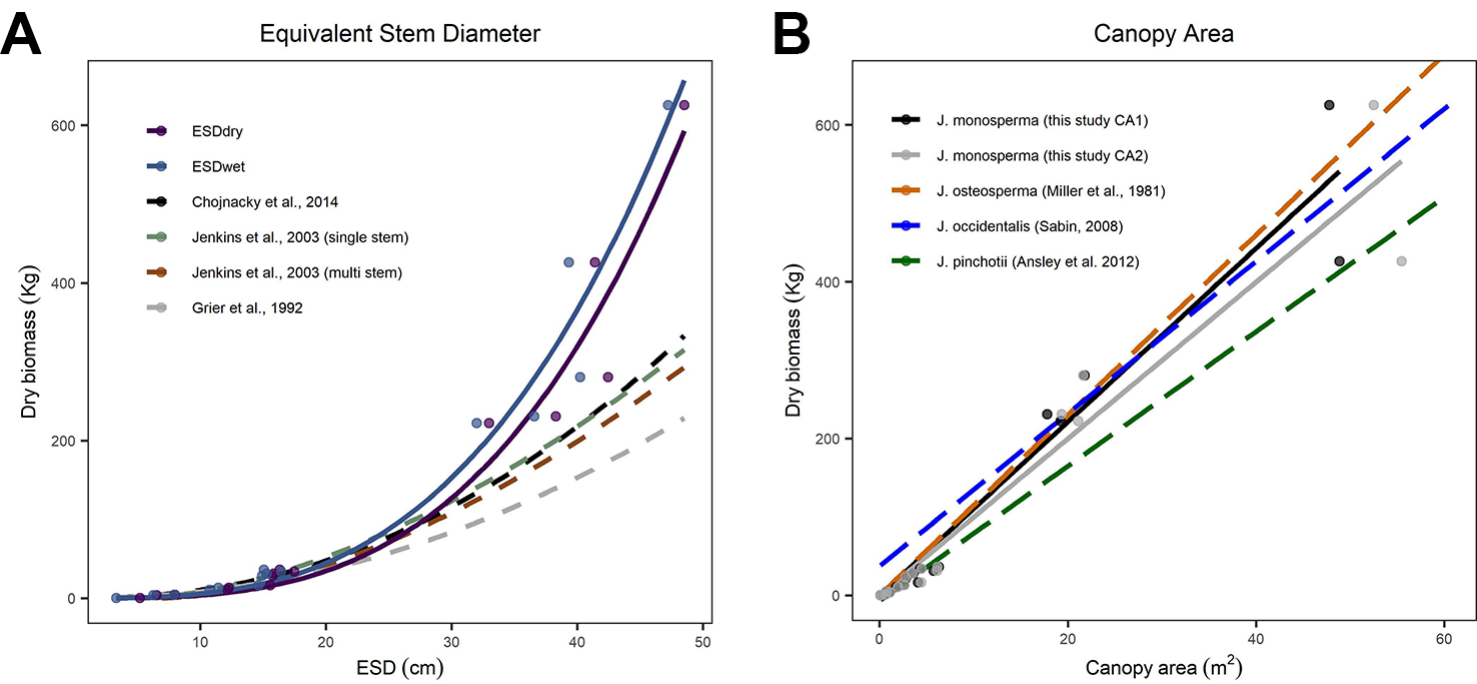
Comparisons between allometric models for predicting aboveground biomass (AGB). **(C)** considers equivalent stem diameter (ESD) as the predictor using the power models fitted to our observations [solid lines, from panel **(A)**] and published models (dashed lines, after Grier et al., 1992; Jenkins et al., 2003; Chojnacky et al., 2014, including both the single-stem and multi-stem versions of the DRC-DBH conversion after Chojnacky and Rogers, 1999). **(B)** considers canopy area as the predictor; the solid lines are the linear models fitted to CA_1_ and CA_2_ [from **Figure 1B**] and the dashed lines are linear models reported for Juniperus occidentalis (Sabin, 2008) and Juniperus pinchotii (Ansley et al., 2012) and fitted to observations of Juniperus osteosperma (Miller et al., 1981).

We compared our observations and fitted models to existing allometric relationships predicting AGB from stem diameter (**Figure 2A**). We found good correspondence between models for the smaller individuals, which had ESD <20 cm and total dry mass <36 kg. However, all three existing models substantially underpredict mass for larger individuals with ESD >30 cm (**Figure 2A**). We also compared our observations and fitted models to existing linear allometric relationships which predict AGB from CA reported for other *Juniperus* species (**Figure 2B**).

### Sapwood Area Relationships

**Figure 3:**
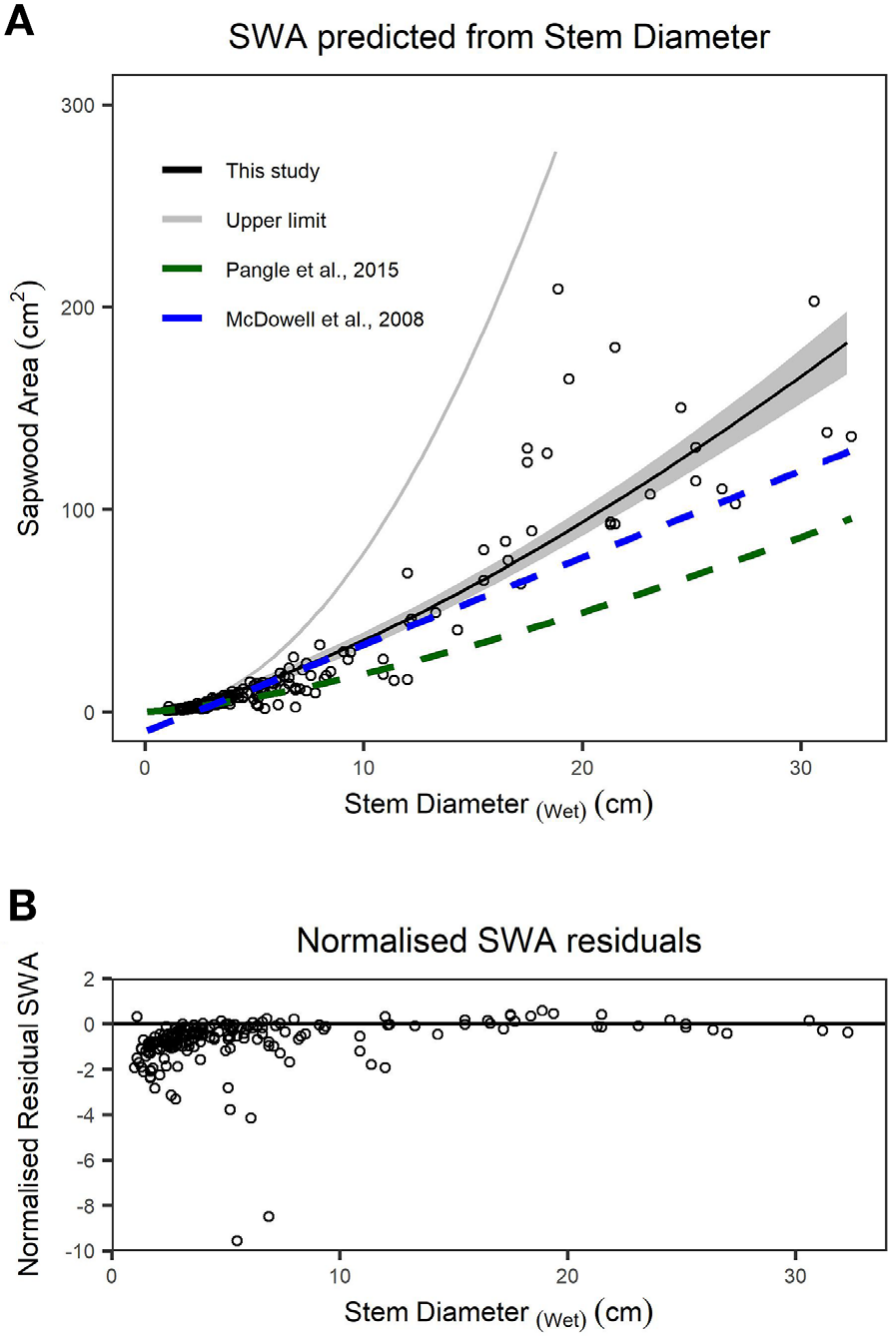
**(A)** Relationship between sapwood area (SWA) and wet stem diameter for individual stems. n = 200 observations, which are described by a power function. The upper limit (grey line) is the area of a circle calculated from diameter at the root collar (DRC) wet and offers an upper bound on the plausibility of SWA models/observations. The dashed lines represent previously published functions for Juniperus monosperma (McDowell et al., 2008b; Pangle et al., 2015). **(B)** The normalized residual SWA as a function of stem diameter. Model parameters and standard errors are provided in **Table 2**.”

We found stem diameter was a strong predictor of SWA (**Figure 3**), with the relationship described by a power function (**Table 2**). The mean normalized residual from our SWA model is -0.75, with a standard deviation (SD) of 1.13, minimum of -9.57 and maximum of 0.58 (**Figure 3B**). We compared our fitted models with published allometries relating diameter to SWA (**Figure 3A**). While ESD can be used to predict tree-level SWA (**Table 2**), the nonlinear relationship between SWA and stem diameter means that the distribution of diameters is important and consequently the best predictions of tree-level SWA are obtained by summing the predicted SWA of each stem.

The relationship between CA and maximum height of *J. monosperma* was curvilinear, with the rate of height increase declining with increasing CA (**Figure S1A**). The relationship between mean canopy diameter and maximum height indicates an increasing deviation from the 1:1 line (i.e., toward an oblate ellipsoid canopy form) with increasing canopy size (**Figure S1B**).

### Measurement of Canopy Area, Equivalent Stem Diameter, and Basal Area

**Figure 4:**
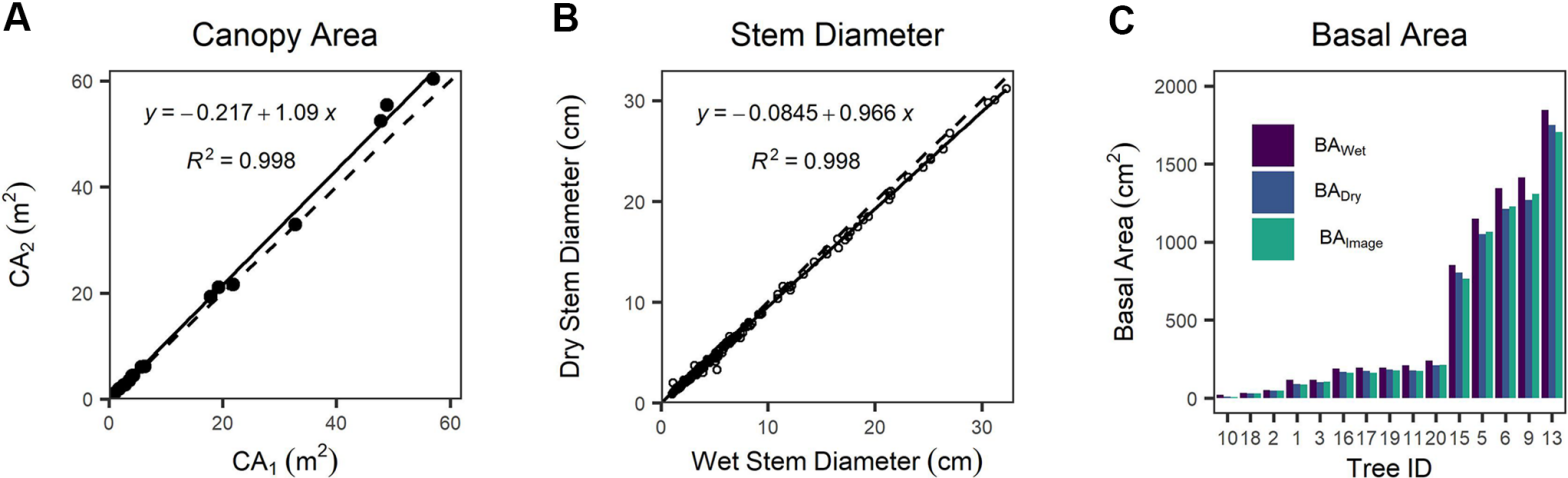
**(A)** Comparison of canopy area (CA)_1_ versus CA_2_ as different measurements of canopy area, **(B)** comparison between wet (field) versus dry (lab) measurements of stem diameter, and **(C)** comparison between basal area measured *via* image analysis (BA_Image_) of dry disks and basal area calculated from the equivalent stem diameter (ESD) of the wet (BA_Wet_) and dry wood (BA_Dry_). In **(A**, **B)** dashed lines represent the 1:1 relationship, and solid lines are fitted linear regression models.

We found a systematic bias between CA derived from detailed tracing around canopy edges shown in the orthomosaic image (CA_1_) and CA computed from canopy length and width (CA_2_) (**Figure 4A** and **Figure S4**). CA_2_ is positively biased with respect to CA_1_ by 8.5%, a statistically clear difference (one-way Wilcoxon signed-rank test with continuity correction, *n* = 20, *v* = 29, *p* = 0.0024). We found cross-sectional disk diameters decreased by 5% on average following drying in the laboratory compared with wet measurements in the field (**Figure 4B**), and that this reduction was statistically clear (one-way paired t-test, df = 198, *t* = 12.833, *p* < 0.0001). BA values differed substantially between measurement techniques (**Figure 4C**). BA computed from ESD_Dry_ was 13.3% smaller than BA computed from ESD_Wet_, and even excluding the JH-10 outlier (the smallest, single-stemmed individual) BA_Dry_ was 10.0% smaller than BA_Wet_. The BA_Image_ was found to be on average 0.1% less than BA_Dry_; however, the single stem of JH-10 was again a significant outlier with a difference of -28.1% in itself. Discounting this single outlier stem resulted in a mean BA_Image_ that was 1.8% greater than the dried samples measured with diameter tape and calipers. We attribute this minor difference to the taper present in tree stems toward the ground–bole interface. The estimates of BA_dry_ and BA_wet_ assume a circular shape, which will inherently introduce a measurement bias when working with highly irregular stems such as those present in *J. monosperma*. For highly irregular disks, BA_Image_ was typically lower than BA_Dry_ due to invaginations that were included in circumference tape measurements.

### Carbon and Nitrogen Composition

Whole-tree mass-averaged C content was 49.2% ± 0.1%, and nitrogen content was 0.4% ± 0.1%. These C coefficients are essential to calculating C stocks from AGB values, and further details for each component are included in **Table S2**.

## Discussion

We harvested 200 individual stems and 18 entire trees to obtain a comprehensive set of SWA and biomass observations for a wide range of *J. monosperma* tree sizes. In terms of maximum height, maximum mean canopy diameter, and maximum stem diameter, the individuals sampled are representative of the *J. monosperma* population at our site and also of populations reported from Los Alamos (Breshears, 2008; n = 278 individuals), the Sevilleta National Wildlife Refuge (Pangle et al., 2015; n = 714 individuals) and Las Vegas, New Mexico (Marcy Litvak, unpublished data; n = 591 individuals); see **Table S1** for comparison. Our observations increase accuracy and confidence in allometric relationships for predicting AGB and SWA for *J. monosperma*.

## Prediction of Aboveground Biomass

We found multiple dimensional attributes were strong predictors of AGB in *J. monosperma*, consistent with studies of other species in this genus. ESD was a very strong predictor of AGB, and both the wet (field) and dry (lab) observations of stem diameter are described well by power models. Because of the systematic bias between wet and dry ESD measurements (**Figure 4B**), we recommend selecting the appropriate model for the type of diameter observations available.

We found substantial differences between published models predicting AGB from ESD compared with our observations and fitted models (**Figure 2A**). We looked at models published by Grier et al. (1992); Jenkins et al. (2003), and Chojnacky et al. (2014). Chojnacky et al. (2014) was an update to the Jenkins et al. (2003) and incorporated the earlier findings of Grier et al. (1992). We therefore focus on the Chojnacky model as the most recent compilation. We found good correspondence between all five models for the smaller individuals (ESD <20 cm and dry mass <36 kg); however, all three existing models substantially underpredict biomass for all five larger individuals (JH13, JH06, JH09, JH05, JH15) with ESD >30 cm (**Figure 2A**). Our observations extend the calibrated range of ESD, which is important given the small number of existing observations constraining the upper end of this relationship (Chojnacky et al., 2014). The mean error in predicted biomass (mean of (estimated mass - observed mass)/observed mass) for the five larger trees harvested was 51.3%, 36.9%, and 30.6%, respectively, for the Grier et al. (1992); Jenkins et al. (2003), and Chojnacky et al. (2014) models. The divergence is substantial (ca. 100%) for the two heaviest trees harvested. We expected some discrepancy given that Chojnacky et al. (2014) highlighted the “woodland Cupressaceae” taxon (consisting of mostly juniper species) as having particularly low confidence, with typical residuals of ±30%. Based on our analysis, we conclude that the Chojnacky et al. (2014) model is not suitable for use with large (≥25 cm ESD) *J. monosperma*.

Moving beyond this particular species and geographic location, our findings address the call by Chojnacky et al. (2014) for new empirical observations describing diameter–biomass relationships in the Cupressaceae group. Our findings support the view of Chojnacky et al. (2014) that the current national-scale model is probably underpredicting biomass in this taxon, particularly where stands comprise of older mature trees with high ESD. This contention is further supported by other recent studies of the *Juniperus* genus. The mean diameter and biomass of the four largest *Juniperus occidentalis* trees studied by Sabin (2008) were 62 cm and 624 kg compared with a prediction of 570 kg from the Chojnacky et al., 2014 model (ca. 9% low). The mean diameter and biomass of the five largest *J. occidentalis* trees studied by Tiedemann and Klemmedson (2000) were 63 cm and 741 kg compared with a prediction of 591 kg from the Chojnacky et al., 2014 model (ca. 20% lower). We anticipate that our new observations will help to inform the next generation of continental-scale allometries, supporting more accurate models with better-understood uncertainties (Jenkins et al., 2003; Chojnacky et al., 2014).

We found CA was a very strong predictor of AGB, and the relationship for both estimates of CA (CA_1_ and CA_2_) were described well by linear models (R^2^ = 0.95 and R^2^ = 0.94, respectively). This is useful because CA is the attribute most easily measured from remote sensing (Sabin, 2008; Strand et al., 2008; Starks et al., 2011; Hulet et al., 2014), unlike stem DRC, which is labor intensive and difficult in many *Juniperus* stands (Ansley et al., 2012; Krofcheck et al., 2016). CA is therefore more readily utilized for landscape-scale estimates of biomass stocks (Strand et al., 2008; Hulet et al., 2014). This is the first study reporting the relationship between CA and observed biomass for *J. monosperma*. The simple linear model facilitates upscaling efforts, particularly as it absolves the need to differentiate coalesced canopies of adjacent trees which can introduce large errors (Cunliffe, 2016; Cunliffe et al., 2016a; Krofcheck et al., 2016). Ideally, more trees with coalesced canopies will also be measured for more robust validation of CA as a predictor of biomass across landscapes. However, Starks et al. (2011) found these linear relationships are preserved even when canopies coalesce in stands of *J. virginiana*.

We found that the two different methods for deriving CA gave different results, with CA_2_ providing positively biased estimates of CA by an average of 8.5% compared to CA_1_ (**Figure 4A**)_,_ with implications for scaling up to landscape extents. We consider the CA_1_ measurement to provide a more accurate representation of canopy cover because unlike CA_2_, it can account for heterogeneity in the crown shape, which can be quite diverse in *J. monosperma*. In this experiment, we used image data with a spatial grain of 2 cm, which means that fine-scale differences in CA_1_ could be characterized in a detailed manner. Starks et al. (2011) also reported that ground-based measures of CA (i.e., similar to CA_2_) were 9% larger than remotely sensed CA (i.e., CA_1_) across canopies ranging from ca. 0.25 to 100 m^2^ in stands of *J. virginiana*. This similarity with our assessment further indicates that this bias persists at coarser scales of observation and should be considered when using literature-derived allometric relationships. Image grain will also impact the accuracy with which CA can be derived from spatial image data—and other users must be mindful of the sensitivity of this method to smaller trees, potentially influencing the detection of encroachment patterns across landscapes. For example, Hulet et al. (2014) consider grains of finer than 1 m^2^ unlikely to have a major influence on remotely sensed AGB estimates in juniper-dominated ecosystems over landscape extents, but Starks et al. (2011) suggest that imagery of 0.45 m^2^ spatial resolution is necessary for analyzing *J*. *virginiana*. We suggest that the CA_1_ approach would work similarly well with aerial images having a spatial grain of ca. 2 m or finer.

We found very close agreement between our observations and linear models predicting AGB from CA for *J. monosperma* when compared with others' findings for *J. occidentalis*, *J. osteosperma*, and *Juniperus pinchotii* (**Figure 2B**; **Table 3**). We found 11.1 kg of biomass per m^2^ of CA for *J. monosperma* compared to 11.5 kg for *J. osteosperma* (after Miller et al., 1981), 12.0 *kg* for *J. occidentalis* (Sabin, 2008), and 8.6 kg for *J. pinchotii* (Ansley et al., 2012). Tiedemann and Klemmedson (2000) also found evidence for a strong linear relationship between CA and biomass for *J. occidentalis*. This strong similarity in relationships between several species supports the use of CA as a predictor of biomass across the *Juniperus* genus, at least where growth forms are similar and lateral canopy growth is emphasized over vertical growth (Ansley et al., 2012).

**Table 3:** Published canopy area (CA)–aboveground biomass (AGB) models for other Juniperus species. Where AGB is in kilograms and CA_2_ is in square meters.

Species	Model	R^2^	N	Range of harvested individuals (kg)	Reference
*J. occidentalis*	AGB = 37.51 + 9.71 * CA_2_	0.86	56	50–850	Sabin (2008)
*J. pinchotii*	AGB = -6.81 + 8.58 * CA_2_	0.94	40	9–688	Ansley et al. (2012)
*J. osteosperma*	AGB = 11.494 * CA_2_	0.95	56	12–956	Miller et al. (1981)

Maximum height was also a strong predictor of biomass in *J. monosperma* (**Figure 1D**). *Juniperus* species commonly prioritize lateral over vertical growth, with commonly wide variance in the relationship between height and biomass (Ansley et al., 2012), so we suggest that height-based models should be used with caution. The relationships we observed between both CA and maximum height, and canopy diameter and maximum height (**Figure S1**) were similar to those reported for *J. pinchotii* by Ansley et al. (2012). Once the trees have a dry mass of more than ca. 40 kg, the proportion of biomass present in the <3 cm component decreased from ca. 81% ± 16% (SD) to ca. 44% ± 5% (SD) (**Figure S2**).

### Insights From Sapwood Area Observations

Cross-sectional SWA is a critical parameter for calculating sapflow through plant stems (Köstner et al., 1998; Čermák et al., 2004). SWA is commonly estimated from breast-height stem diameter; however, this relationship is particularly challenging for *Juniperus* species due to their complex multi-stem morphology and also their irregular distribution of SWA around individual stems (McDowell et al., 2008b) (**Figure S3**). We noted that the distribution of sapwood around the axis of disk samples was often asymmetric (see **Figure S3**), such that heartwood and sapwood were often opposing rather than forming a continuous band of sapwood around the heartwood that is typical for single-stemmed tree species. We observed this asymmetry more frequently in larger (>10 cm) disks, and sapwood was consistently concentrated within the portion of the stem facing the nearest edge of the tree (i.e., away from the center mass). The highly irregular sapwood thickness about the stem axis would hinder accurate determination of SWA from a single core sample, a less destructive method typically used to infer the sap conducting area when scaling point transpiration measurements (Looker et al., 2016). While care was taken to obtain a representative DRC measurement with tape or calipers at the midpoint of the disk sample, in some cases the wider portion of the disk was sanded and used for image analysis. We argue that the imaged BA provides a more accurate representation of the actual space occupied by a given stem, since it accounts for the deep grooves and invaginations that are common in *J. monosperma* stems which traditional field measurements would otherwise include as “empty space” (McDowell et al., 2008b) (**Figure S3**). We examined the relationship between diameter and SWA at the stem level using wet diameter measurements for maximum relevance to nondestructive field applications. Our 200 observations were described well by a two-parameter power function (**Figure 3**; **Table 2**), which is consistent with the model form reported for many other tree species (Vertessy et al., 1995; Jung et al., 2011; Brantley et al., 2016; Aparecido et al., 2019).

We found substantial disagreement between our diameter–SWA observations and model relative to those previously reported for *J. monosperma* (**Figure 3**). Two previous studies have reported relationships between diameter and SWA for this species. McDowell et al. (2008b) harvested *n* = 10 trees, ranging from ca. 3 to 20 cm diameter, and fitted a linear model (Eq. 8) which predicts negative SWA for stems smaller than 2.3 cm diameter. Pangle et al. (2015) harvested *n =* 24 branches, ranging from 1.7 to 18.6 cm, and fitted a (near-linear) power model (Eq. 9). Our observations agree with the previously published models for stem diameters smaller than ca. 12 cm, rapidly diverge for stem diameters greater than ca. 12 cm. This improved knowledge of the diameter–SWA relationship for this species helps estimate and scale physiological attributes such as transpiration (McDowell et al., 2008b; Pangle et al., 2015). As the accuracy of whole-tree and stand transpiration estimates relies on robust allometric models for predicting total SWA (Čermák and Nadezhdina, 1998; Kumagai et al., 2005), substantial sources of error may be introduced when using the previously published models to estimate SWA for stems with diameters larger than 14 cm. We reiterate that these allometric models should only be applied within their calibrated ranges. Although ESD can be used to predict SWA (**Table 2**), ESD-based predictions are not recommended because the nonlinear relationship means that the SWA is highly sensitive to the distribution of stem diameters. The best possible estimate of SWA at the tree level is obtained by summing the SWA predicted for each stem. Our SWA normalized residuals are high compared with those typically reported from other species with more uniform diameter–SWA relationships, highlighting the importance of appropriately propagating uncertainty in SWA estimates for transpiration upscaling studies (Looker et al., 2016).

### Carbon and Nitrogen Content

The C content of wood is widely assumed to be 50%; however, few studies explicitly quantify the C content of target species. Previous investigations have revealed C contents can be more variable, especially in softwoods such as *Juniperus* (Lamlom and Savidge, 2003; Cunliffe, 2016). We found whole-tree C content was 49.2% ± 0.1%, and nitrogen content was 0.4% ± 0.1% (**Table S2**). For *J. monosperma*, we know of only a single published C content of 51% based on a single measurement of a <3 cm diameter shoot (Puttock, 2013). Across the genus, others have reported whole-tree values of 48.7% for *J. occidentalis* (Tiedemann and Klemmedson, 2000) and 52% and 50% for *J virginiana* (Norris et al., 2001; Lamlom and Savidge, 2003). Our analysis supports the use of a 50% approximation for *J. monosperma*, although we advocate accounting for the variability in C content to obtain more robust estimates of landscape-level C storage (Cunliffe et al., 2016a).

## Conclusion

Better estimates of ecosystem functions including C storage and transpiration are important for understanding the role of semiarid ecosystems at local, regional, and global scales. These new observations of *J. monosperma* allometry are an important contribution to the literature describing this species, part of the “woodland Cupressaceae” taxon that has previously had poorly constrained allometric functions. Measuring 18 trees with dry masses ranging from 0.4 to 625 kg, we found that ESD was a strong predictor of AGB; however, the relationship increased much more steeply than predicted by existing models, suggesting that existing allometric functions are poorly suited to *J. monosperma*. We found a strong linear relationship between CA and AGB (r^2^ = 0.96), which was similar to those previously reported for other juniper species. This finding supports the application of remote sensing approaches based on CA alone to measure and monitor changes in AGB stocks in juniper-dominated ecosystems, although it is necessary to account for biases of 9% when using different approaches of measuring juniper CA. Stem diameter predicted SWA well at the level of individual stems, although the relationship is quite variable for this species. Critically, we found a power relationship between stem diameter and SWA, which indicates tree-level SWA will be more accurately estimated by summing the SWA predictions from individual stems, rather than using functions based on ESD. These better-constrained allometric models for *J. monosperma* will support more accurate and robust estimates of both C storage and transpiration fluxes in *Juniperus*-dominated ecosystems.

## Data Availability Statement

The datasets presented in this study are available from the NERC Environmental Information Data Centre (DOI: 10.5285/871443a9-6634-4eba-abb5-286a1ab58e9b), and the raw data supporting the conclusions of this article will be made available by the authors, without undue reservation, to any qualified researcher.

## Ethics Statement

Written informed consent was obtained from the individual for the publication of any potentially identifiable images included in this article.

## Author Contributions

This study was conceptualized by AC and CM. Funding was acquired by RB, AC, KA, and ML. The methodology was planned by AC and CM. Investigation was undertaken by AC, CM, FB, KA, and KS. Formal analysis, visualization, and project administration were undertaken by AC. Data are curated by AC and CM. The original draft was written by AC and was revised with input from all authors.

## Funding

This study was part of the DRIVING-C project funded by the U.K. Natural Environment Research Council (NE/R00062X/1) awarded to RB, AC, and KA, and the U.S. National Science Foundation (DEB #1557262) awarded to ML.

## Conflict of Interest

The authors declare that the research was conducted in the absence of any commercial or financial relationships that could be construed as a potential conflict of interest.
