# Supplementary material for: Allometric Relationships for Predicting Aboveground Biomass and Sapwood Area of Oneseed Juniper (Juniperus monosperma) Trees
Source: Front Plant Sci. 2020 Feb 26;11:94. doi: 10.3389/fpls.2020.00094 (PMC7054341; doi:10.3389/fpls.2020.00094)

Supplementary Information

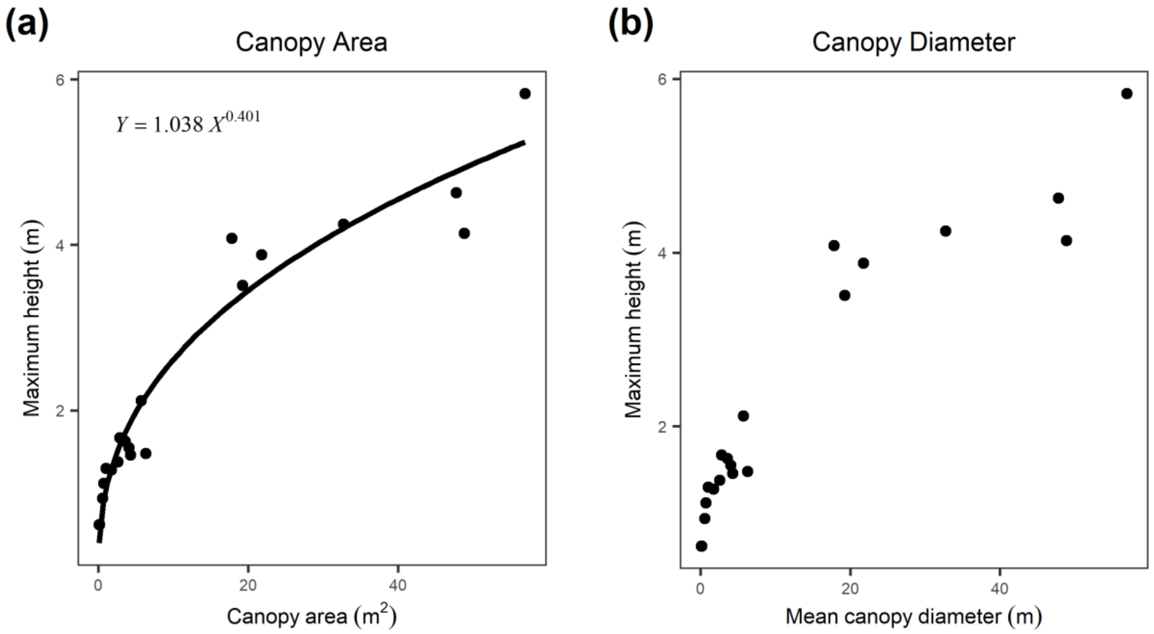

Figure S1. Relationship between (a) maximum tree height and canopy area, and (b) maximum tree height and average canopy diameter (mean of the two canopy diameter measurements per tree) for individual J. monosperma trees in central New Mexico, USA.

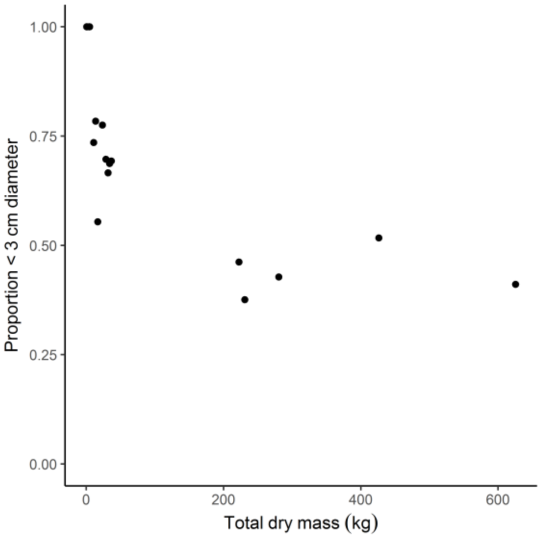

Figure S2. Proportion of biomass < 3 cm diameter by dry total mass.

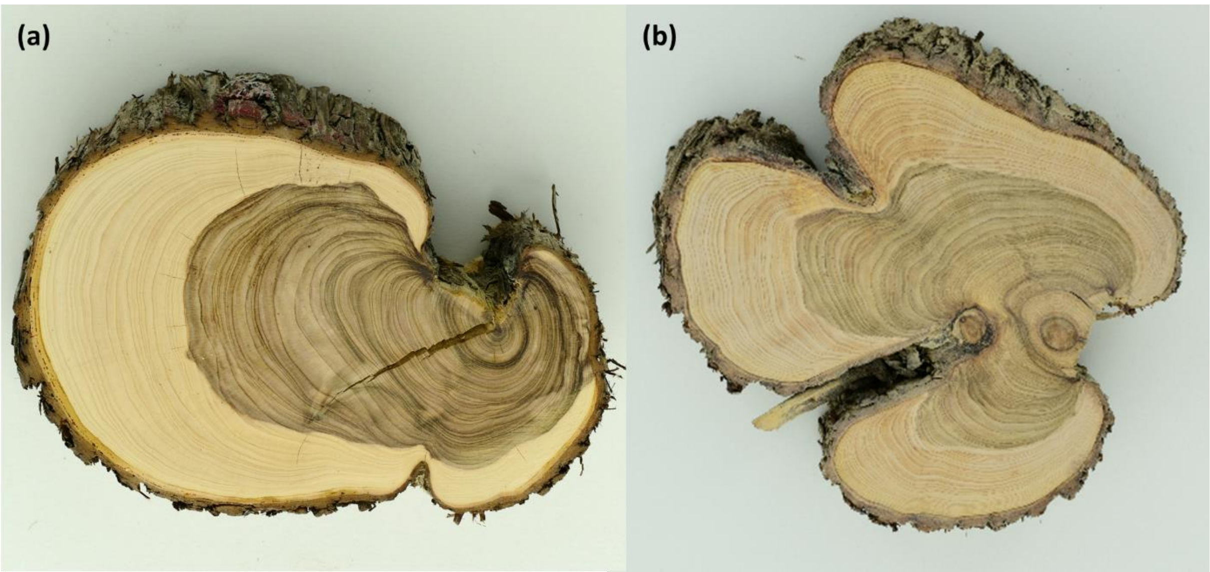

Figure S3. Example cross-section disks from trees JH05 (a) JH15 (b), illustrating the highly variable distribution of the (lighter) sapwood around the (darker) heartwood.

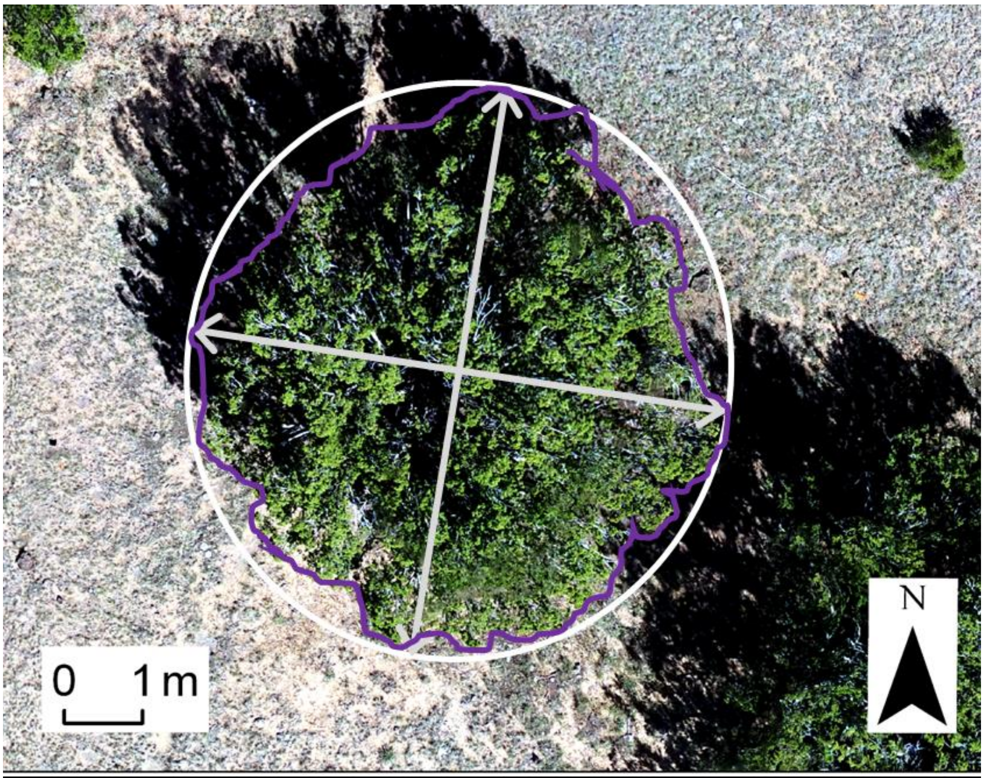

Figure S4. Example of the canopy area (CA) determined for tree JH14 by digitally tracing around the edge of the canopy in the orthomosaics in a GIS environment (CA_1, shown in purple), versus the area of an ellipse constrained by the lengths of the longest and perpendicular axes (CA_2, shown in white). Over all samples, CA_2 was positively biased by 8.5% relative to CA_1.

Table S1. Comparison of maximum dimensions reported for *J. monosperma*.

**Site name**	**WJS, New Mexico**	**Los Alamos, New Mexico**	**Sevilleta LTER, New Mexico**	**Las Vegas, New Mexico**
**Reference**	This study – harvested	Breshears, 2008	Pangle et al., 2015	Marcy Litvak, unpublished data
Maximum tree height	4.6 m	5.6 m	NA	8.3
Maximum mean canopy diameter	8.2 m	6.5 m	NA	NA
Maximum equivalent stem diameter	49 cm	62 cm	70 cm	46 cm
Number of individuals	18	278	714	591

Table S2. Carbon and nitrogen contents for aboveground biomass

**Plant component**		**Carbon content**		**Nitrogen content**		**C:N Ratio**
		**Mean %**	**SD %**	**Mean %**	**SD %**	
< 3 cm diameter		49.338	0.690	0.601	0.091	82.11
> 3 cm diameter		48.928	0.442	0.237	0.073	206.74
Whole-tree mass-weighted mean	All trees	49.215	0.091	0.496	0.081	99.32
	Largest five trees	49.108	0.022	0.397	0.020	123.77

SD = Standard deviation.

Table S3. Tree-level observations for all individuals.

Individual	N of stems	Dry mass [kg]			Water content of wet mass [%]			Canopy dimensions				BA_{Image} [cm^2]	Sapwood area [cm^2]
		**< 3 cm component**	**> 3 cm component**	**Total**	**< 3 cm component**	**> 3 cm component**	**Total**	**Canopy Area (CA1) [m^2]**	**Maximum length [m]**	**Perpendicular width [m]**	**Maximum Height [m]**		
JH01	14	7.86	2.83	**10.69**	0.426	0.355	0.409	1.74	1.68	1.46	1.28	88.7	54.2
JH02	18	4.64	0.00	**4.64**	0.424	NA	0.424	0.72	1.04	0.94	1.12	48.9	32.0
JH03	21	10.54	2.90	**13.45**	0.456	0.448	0.454	2.57	1.89	1.78	1.38	106.1	73.6
JH04	NA	0.43	0.00	**0.43**	0.618	NA	0.618	0.15	0.48	0.42	0.62	NA	NA
JH05	12	86.88	144.14	**231.02**	0.407	0.248	0.317	17.81	5.51	4.47	4.08	1066.0	374.9
JH06	9	220.35	205.94	**426.29**	0.388	0.414	0.401	48.86	9.63	7.33	4.14	1226.5	741.3
JH07	NA	18.04	5.24	**23.29**	0.446	0.360	0.429	2.82	2.01	1.76	1.67	NA	NA
JH08	NA	1.45	0.00	**1.45**	0.514	NA	0.514	0.56	0.97	0.86	0.94	NA	NA
JH09	12	120.14	160.34	**280.48**	0.443	0.371	0.404	21.78	5.58	4.93	3.88	1307.3	720.5
JH10	1	0.36	0.00	**0.36**	0.315	NA	0.315	0.09	0.41	0.30	0.62	5.1	3.0
JH11	15	25.25	11.18	**36.43**	0.453	0.380	0.433	6.29	2.89	2.71	1.48	175.0	109.5
JH12	NA	NA	NA	**NA**	NA	NA	NA	56.95	9.51	8.09	5.83	NA	NA
JH13	5	257.33	368.22	**625.55**	0.363	0.270	0.311	47.79	8.93	7.49	4.63	1703.6	398.2
JH14	NA	NA	NA	**NA**	NA	NA	NA	32.72	6.68	6.29	4.25	NA	NA
JH15	11	102.70	119.70	**222.40**	0.405	0.306	0.356	19.23	5.49	4.90	3.51	765.9	276.4
JH16	14	9.16	7.37	**16.54**	0.419	0.368	0.397	4.06	2.43	2.31	1.55	161.2	83.5
JH17	14	19.88	8.64	**28.52**	0.474	0.393	0.452	3.57	2.20	2.03	1.63	162.3	117.3
JH18	7	4.08	0.00	**4.08**	0.436	NA	0.436	1.015	1.25	1.13	1.3	30.9	21.1
JH19	11	21.00	10.55	**31.55**	0.435	0.373	0.416	5.706	2.95	2.63	2.12	176.1	115.8
JH20	26	23.37	10.63	**34.00**	0.402	0.376	0.394	4.278	2.45	2.32	1.46	214.0	151.6

*BA_{Image} and sapwood area were determined from image analysis.

Table S4. Photographs of harvested individuals.

Individual	**Ground Image 1**	**Ground Image 2** (perpendicular to image 1)	**Aerial Image (from orthomosaic)**
JH01	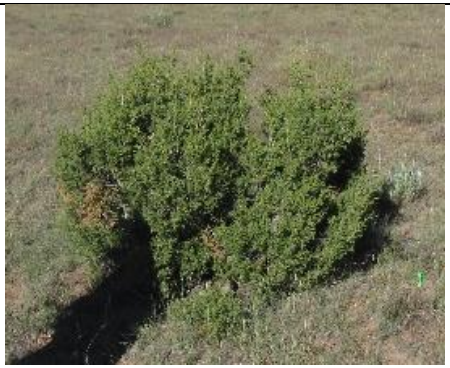	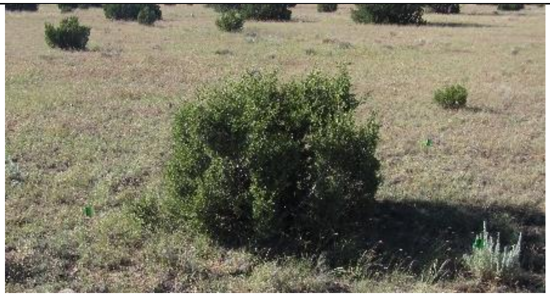	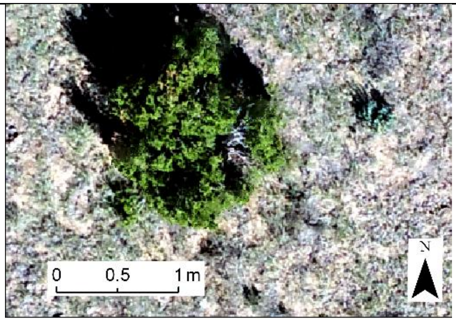
JH02	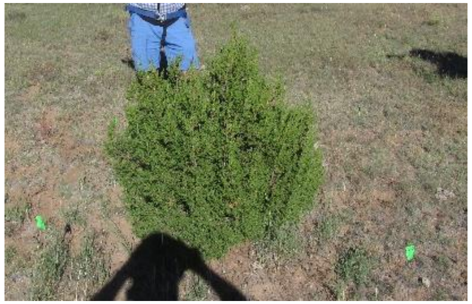	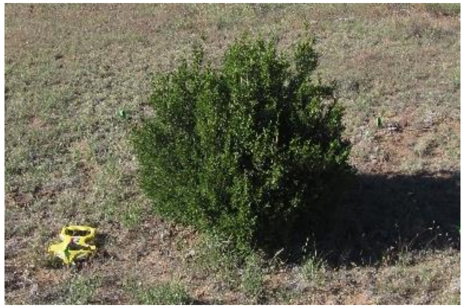	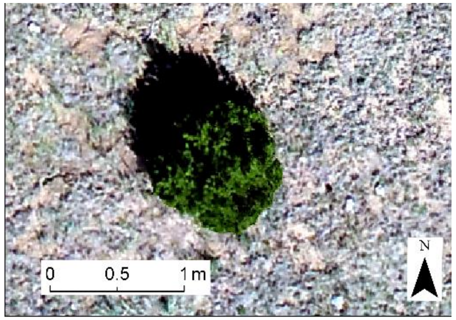
JH03	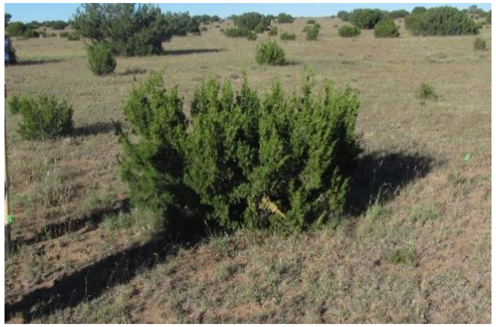	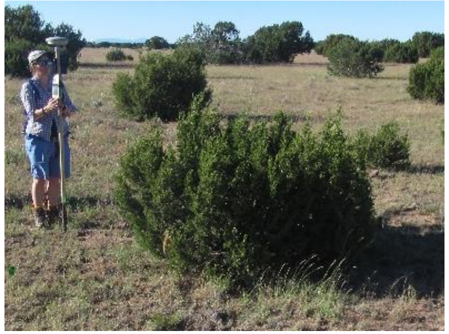	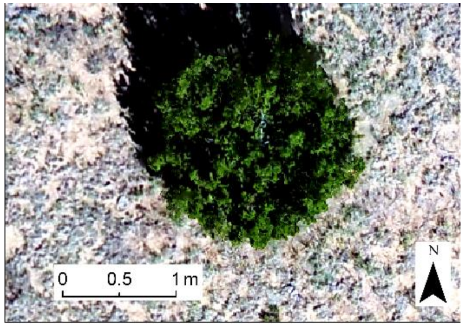

JH04

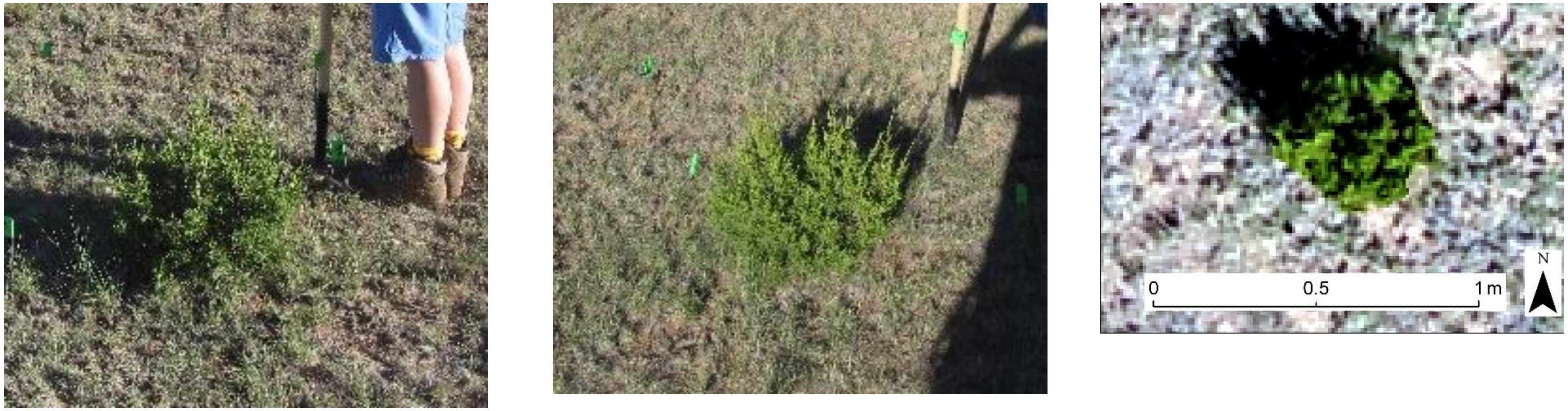

JH05

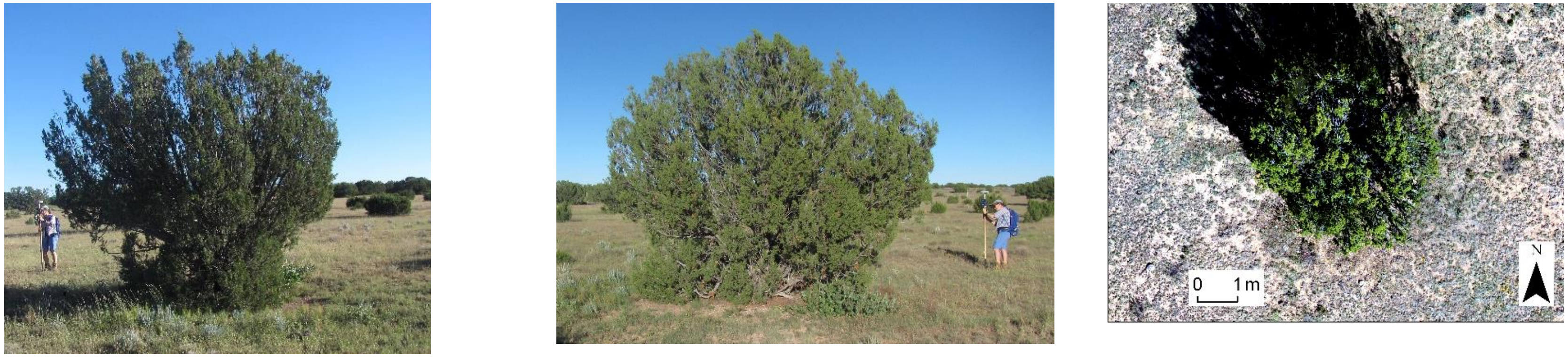

JH06

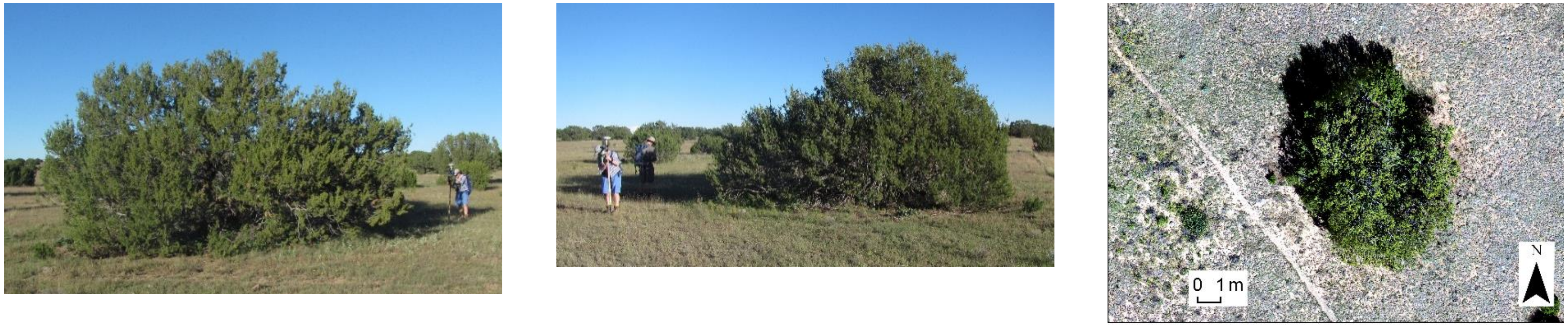

JH07

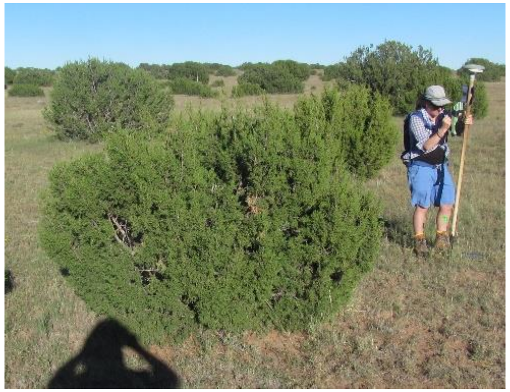

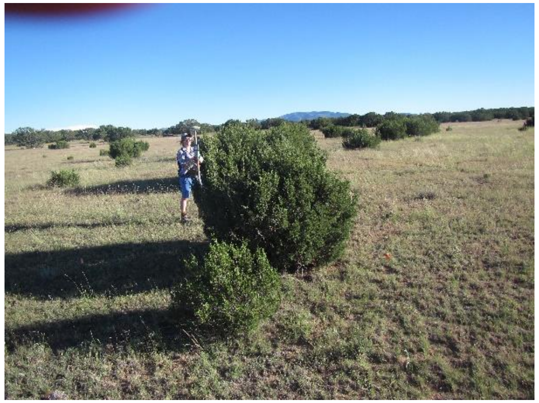

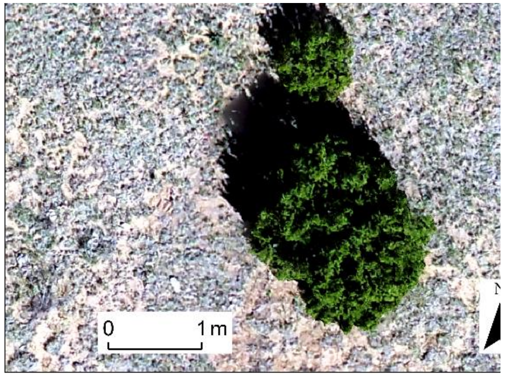

JH08

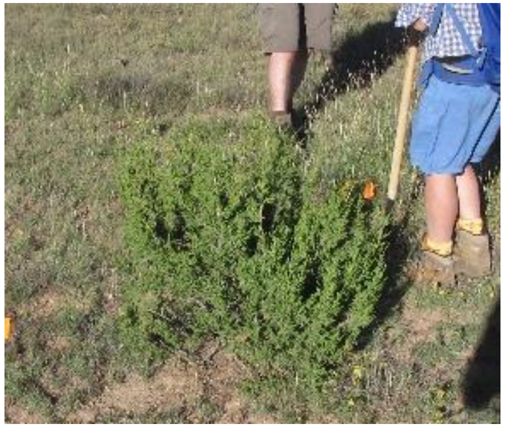

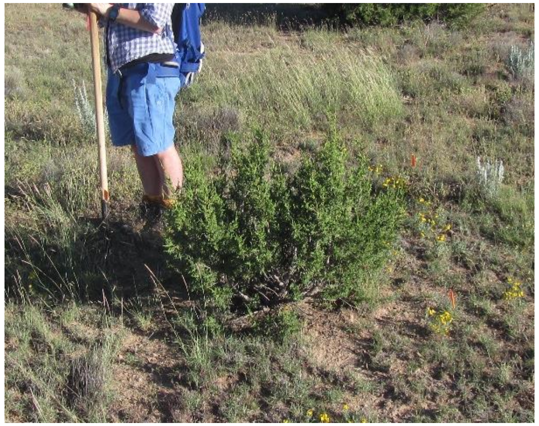

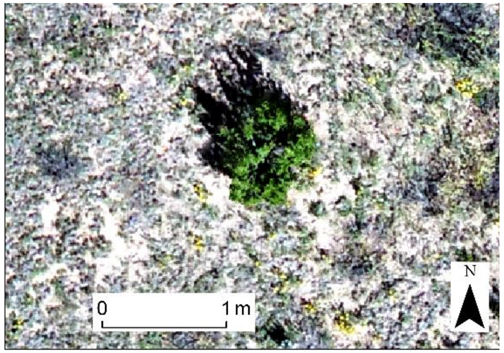

JH09

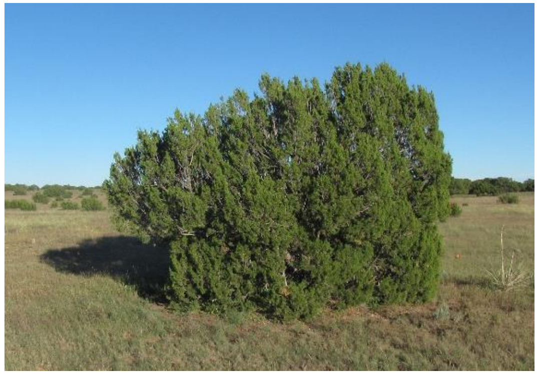

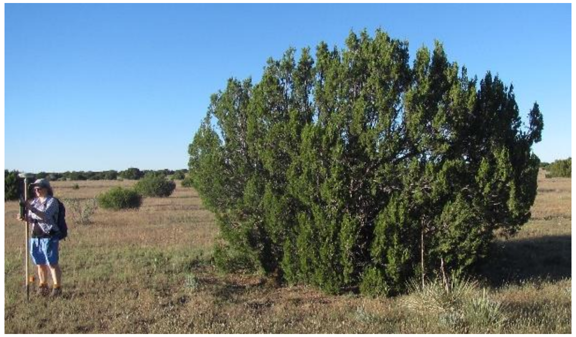

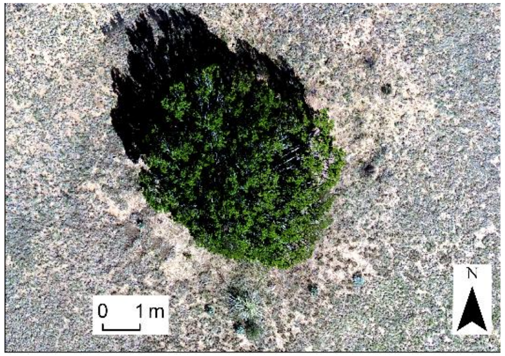

JH10

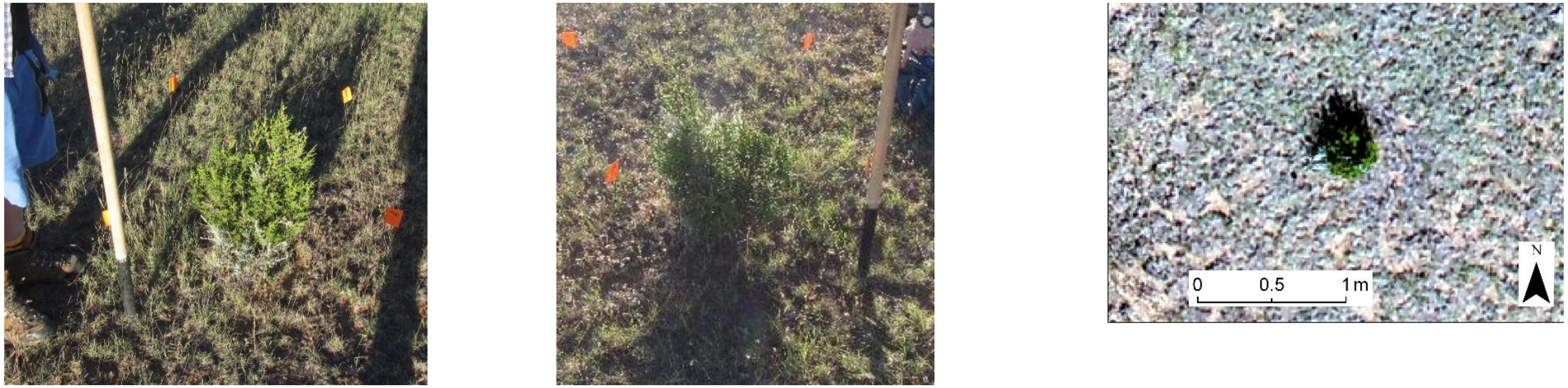

JH11

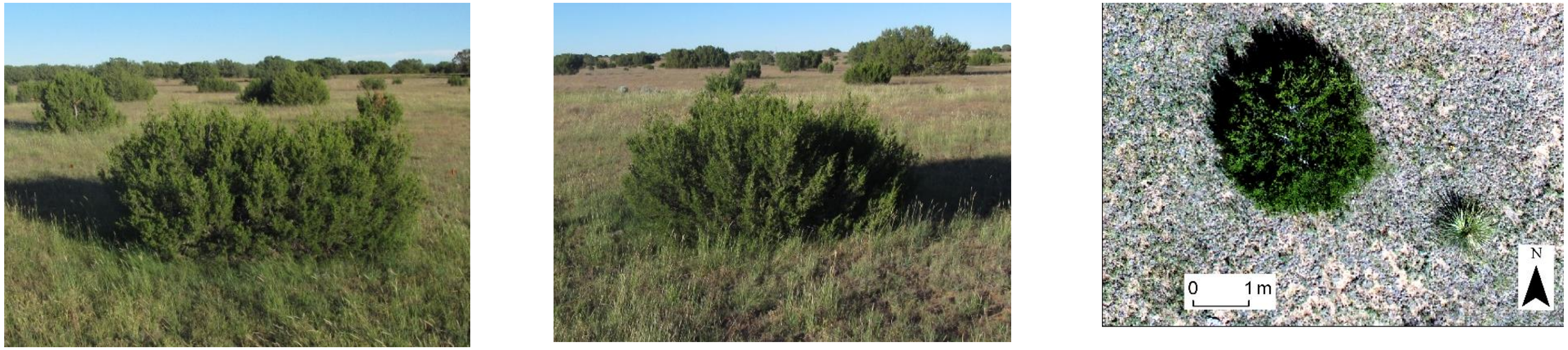

JH12

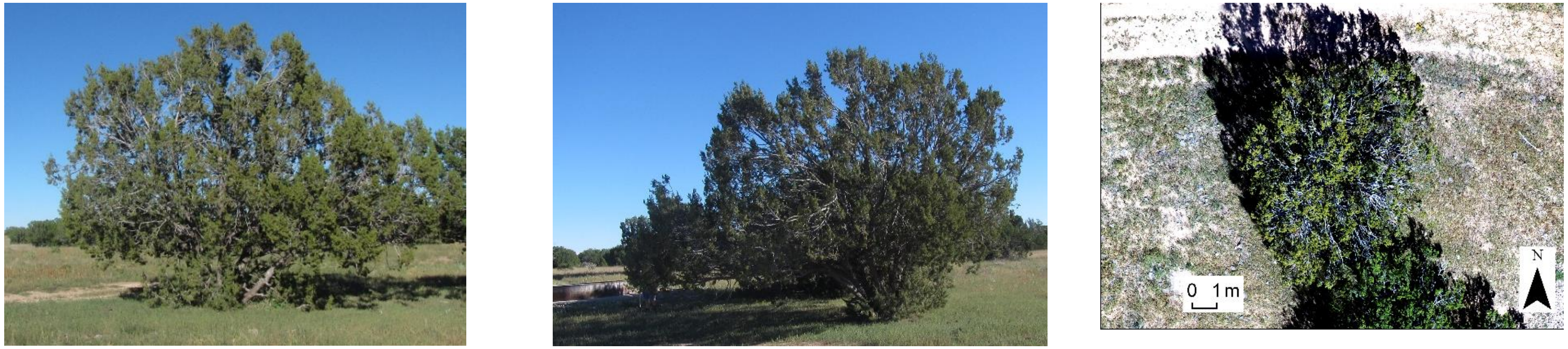

JH13

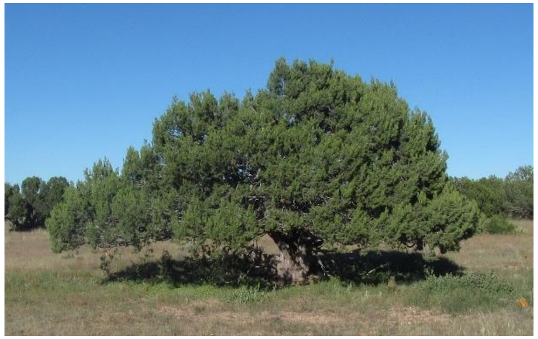

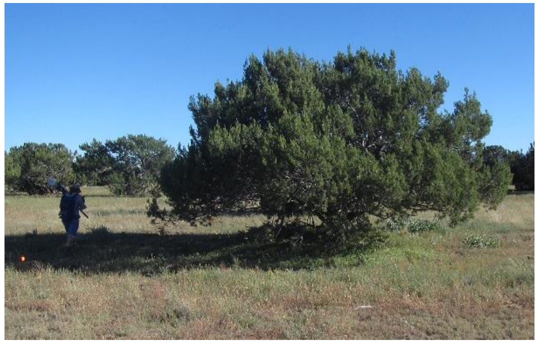

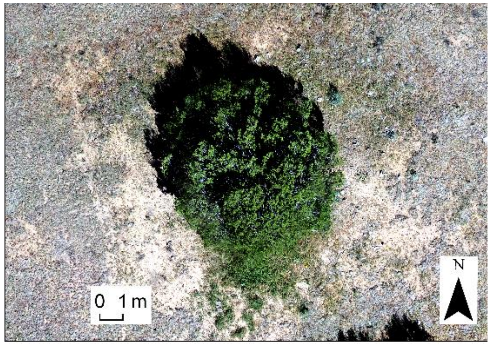

JH14

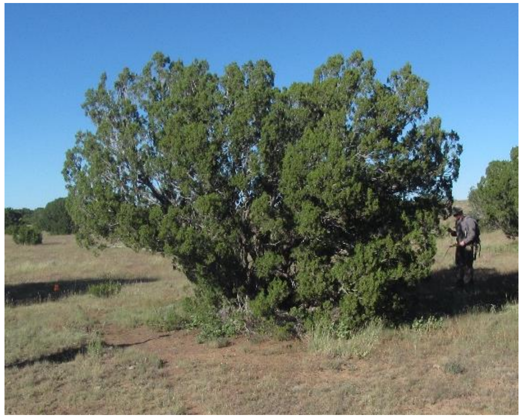

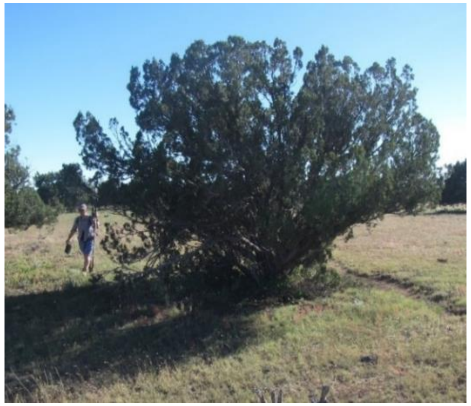

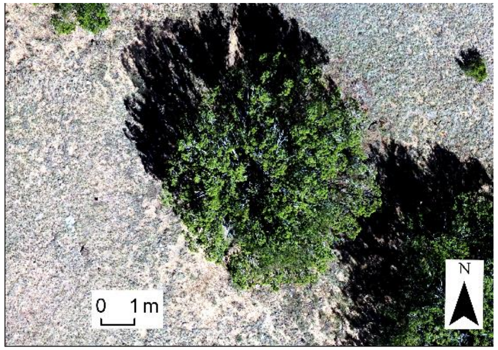

JH15

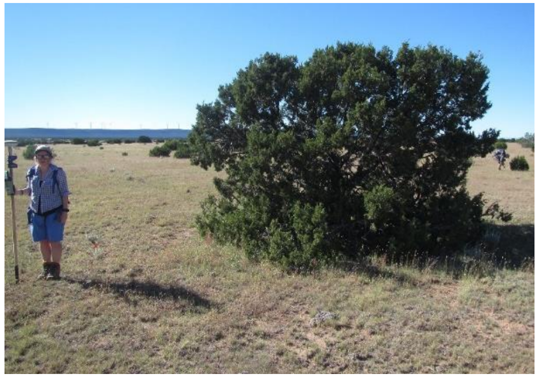

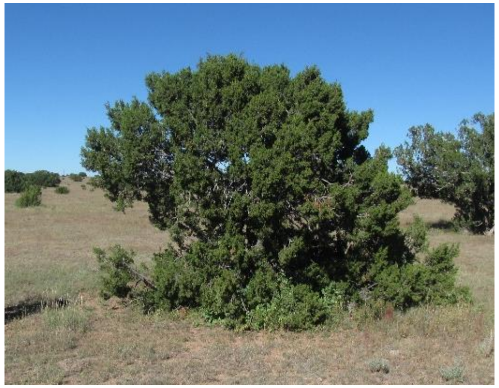

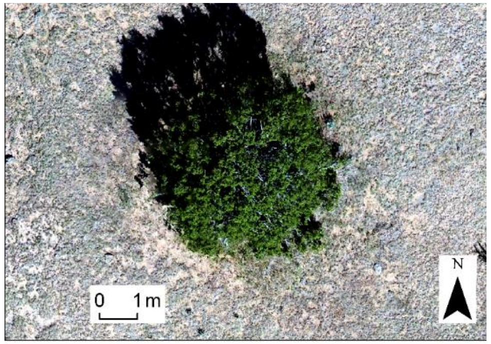

JH16

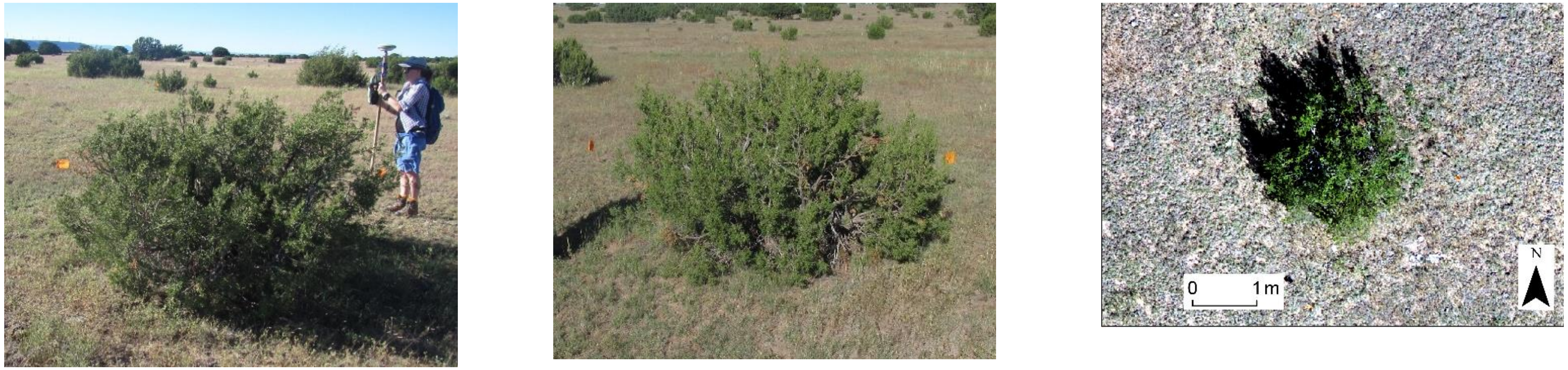

JH17

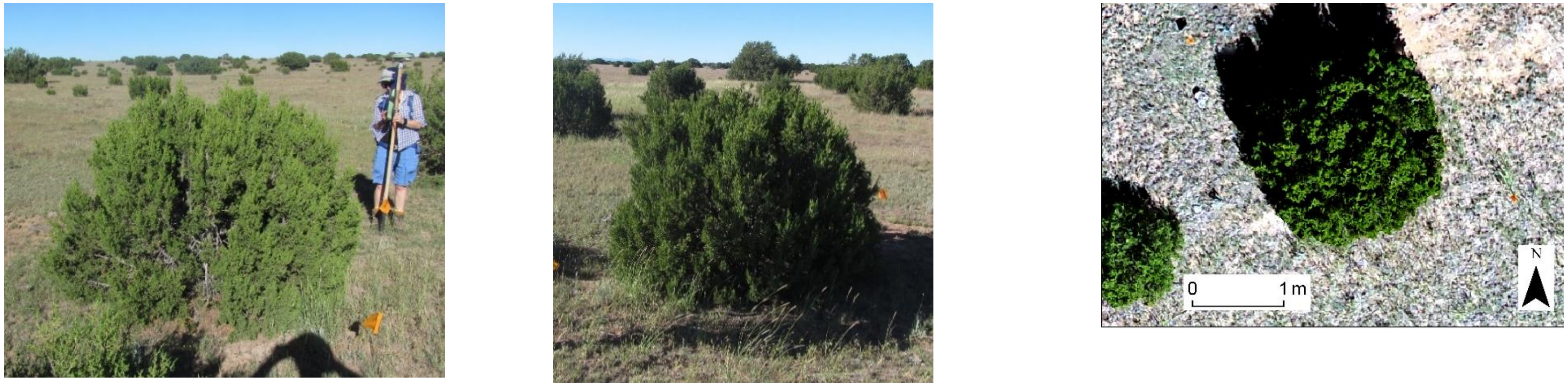

JH18

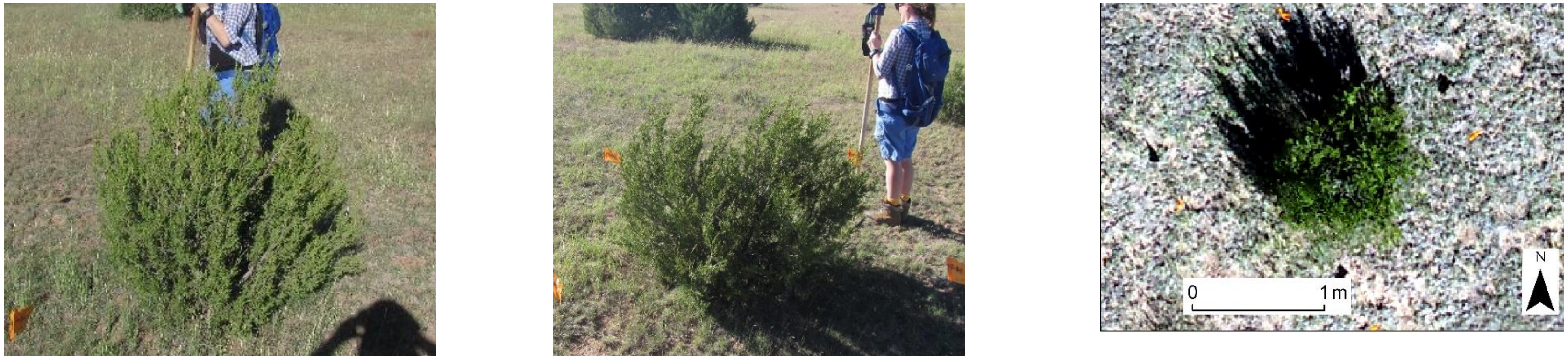

JH19

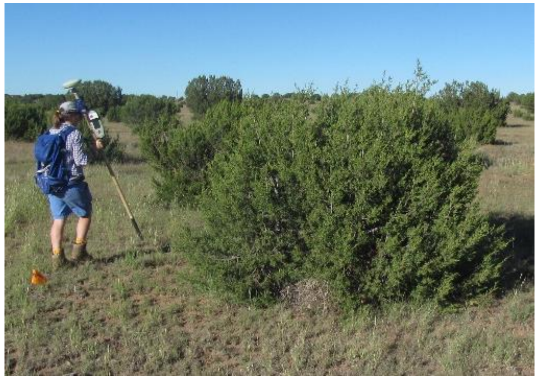

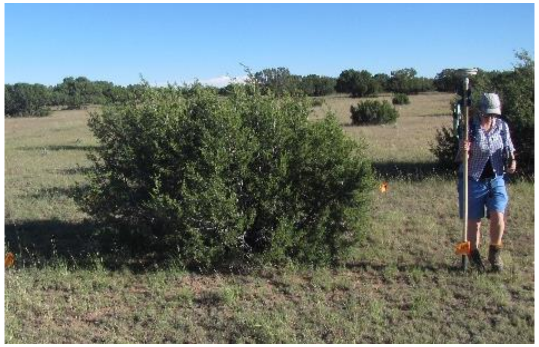

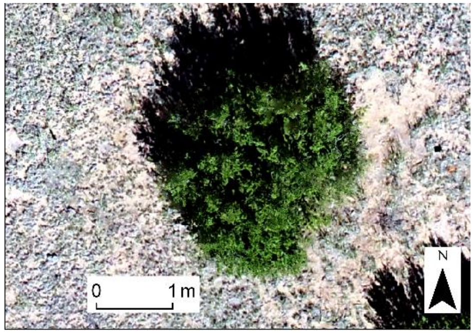

JH20

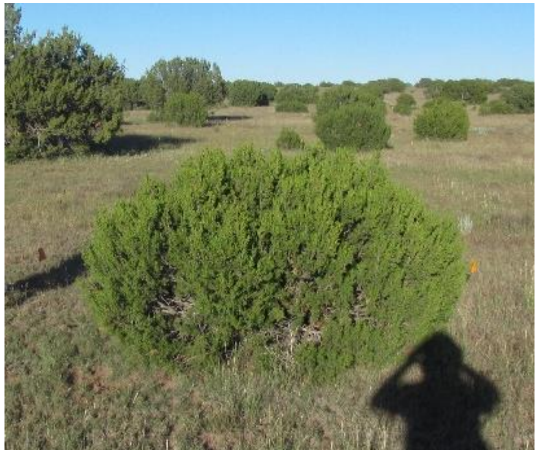

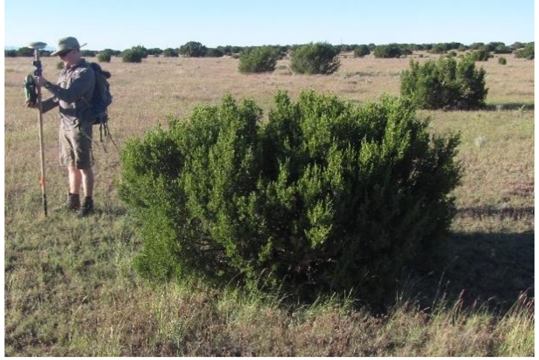

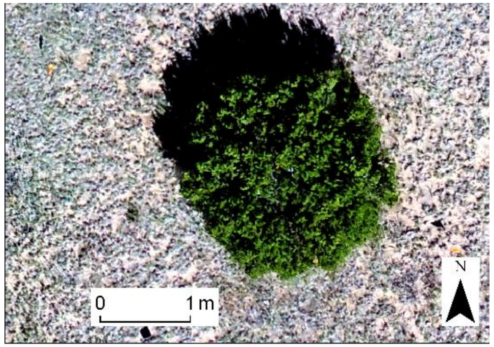

Supplement: Supplementary file 1 [file DataSheet_1.pdf]
